# The effect of the timing of exposure to *Campylobacter jejuni* on the gut microbiome and inflammatory responses of broiler chickens

**DOI:** 10.1186/s40168-018-0477-5

**Published:** 2018-05-12

**Authors:** Phillippa L. Connerton, Philip J. Richards, Geraldine M. Lafontaine, Peter M. O’Kane, Nacheervan Ghaffar, Nicola J. Cummings, Darren L. Smith, Neville M. Fish, Ian F. Connerton

**Affiliations:** 1 Division of Food Sciences, School of Biosciences, Sutton Bonington Campus, University of Nottingham, Loughborough, Leicestershire LE12 5RD UK; 2 Applied Sciences, University of Northumbria, Newcastle upon Tyne, Nothumbria, NE1 8ST UK; 3 Dairy Crest Ltd, Claygate House, Littleworth Road, Esher, Surrey KT10 9PN UK

**Keywords:** *Campylobacter jejuni*, Chicken gut microbiota, Intestinal cytokine and chemokines, Pro-inflammatory response, Gut histology, Food safety

## Abstract

**Background:**

Campylobacters are an unwelcome member of the poultry gut microbiota in terms of food safety. The objective of this study was to compare the microbiota, inflammatory responses, and zootechnical parameters of broiler chickens not exposed to *Campylobacter jejuni* with those exposed either early at 6 days old or at the age commercial broiler chicken flocks are frequently observed to become colonized at 20 days old.

**Results:**

Birds infected with *Campylobacter* at 20 days became cecal colonized within 2 days of exposure, whereas birds infected at 6 days of age did not show complete colonization of the sample cohort until 9 days post-infection. All birds sampled thereafter were colonized until the end of the study at 35 days (mean 6.1 log_10_ CFU per g of cecal contents). The cecal microbiota of birds infected with *Campylobacter* were significantly different to age-matched non-infected controls at 2 days post-infection, but generally, the composition of the cecal microbiota were more affected by bird age as the time post infection increased. The effects of *Campylobacter* colonization on the cecal microbiota were associated with reductions in the relative abundance of OTUs within the taxonomic family *Lactobacillaceae* and the *Clostridium* cluster XIVa. Specific members of the *Lachnospiraceae* and *Ruminococcaceae* families exhibit transient shifts in microbial community populations dependent upon the age at which the birds become colonized by *C*. *jejuni*. Analysis of ileal and cecal chemokine/cytokine gene expression revealed increases in IL-6, IL-17A, and Il-17F consistent with a Th17 response, but the persistence of the response was dependent on the stage/time of *C*. *jejuni* colonization that coincide with significant reductions in the abundance of *Clostridium* cluster XIVa.

**Conclusions:**

This study combines microbiome data, cytokine/chemokine gene expression with intestinal villus, and crypt measurements to compare chickens colonized early or late in the rearing cycle to provide insights into the process and outcomes of *Campylobacter* colonization. Early colonization results in a transient growth rate reduction and pro-inflammatory response but persistent modification of the cecal microbiota. Late colonization produces pro-inflammatory responses with changes in the cecal microbiota that will endure in market-ready chickens.

**Electronic supplementary material:**

The online version of this article (10.1186/s40168-018-0477-5) contains supplementary material, which is available to authorized users.

## Background

The production of poultry for both meat and eggs has been increasing rapidly throughout the world [1]. Feed conversion efficiency is of foremost importance in the economic profitability of poultry meat production, and selective breeding has resulted in fast-growing birds with reduced feed conversion ratios. The relationship between the gut microbiota and the feed conversion performance of broiler chickens has been a focus of research in recent years, with the prospect of modifying the microbiota to improve production efficiency and bird health [2, 3].

Food-borne enteritis caused by the Gram-negative spiral-shaped bacteria *Campylobacter* is a major medical and economic problem worldwide, with numbers of cases continuing to increase [4]. Poultry products are considered to be a significant source of infection to humans [5]. *Campylobacter jejuni* and *coli*, the two species responsible for most human disease, are extremely prevalent in poultry production with up to 80% of flocks harboring the bacteria (depending on the country in question), and this leads to a similarly high level of transference to poultry meat following processing [6, 7]. Consequently, much attention has focused on reducing both the incidence of *Campylobacter* in poultry flocks and the numbers of the bacteria contaminating poultry meat and thereby reducing the risk of infection to the consumer. One approach is to attempt to influence the microbiota of the gastrointestinal tract (GI). The development of affordable next-generation DNA sequencing techniques has allowed detailed investigations into the diversity of this important ecosystem and offered the possibility of relating changes in the microbiota to bird health and the efficiency of feed digestion [3].

Once hatched, the GI of chicks becomes successively colonized by *Enterobacteriaceae* (1 to 3 days of age) and *Firmicutes* (approximately 7 days of age onwards) [8]. In the absence of deliberate population of the gut with commercial microbiota preparations, colonization of the avian GI tract with specific bacterial species, belonging to the *Enterobacteriaceae* or *Firmicutes* groups, is likely a stochastic process driven by exposure to bacteria from the rearing environment (which may or may not contain *Campylobacter*) and from bacteria present in food and water. Commercial broiler chickens are typically reared in barns containing flocks of 20,000 birds or more. Chickens are coprophagic, and under commercial conditions, successful intestinal colonizing microorganisms can be dispersed rapidly throughout the flock and represent a significant source of microbiota to other flocks on the farm. *Campylobacter* is usually detected at around 3 weeks of age but rarely in younger birds. At this stage, *Campylobacter* is an efficient colonizer with the frequency of colonization increasing from 5 to 95% within 6 days [9]. This suggests that *Campylobacter* becomes “transmissible” at around 2 weeks. The question arises as to what happens with regard to *Campylobacter*, during the first 2 weeks of life, the so called lag period. It has been shown that chickens aged between 0 and 3 days of age can become infected and shed *Campylobacter* [10]. However, since the occurrence of a “lag period” is frequent, flock level evidence for early infection and shedding is limited [11]. It has been speculated that maternal antibodies provide protection from colonization by *Campylobacter* during the first 2 weeks of life but decline thereafter [12, 13]. The mechanism behind this resistance would be by prevention of proliferation of *Campylobacter* cells in the GI, rather than a specific bacteriocidal action. This might involve competition with, or inhibition by, the resident microbiota in conjunction with the immune system [14]. However, mathematical models of *Campylobacter* transmission support the contention that an age-dependent mechanism is responsible for the lag period rather than any change in susceptibility [15]. Understanding the temporal influence of *Campylobacter* colonization of broiler chickens will provide insight into impact on production parameters and has the potential to reveal strategies to reduce viable numbers on finished product and improve food safety.

Researchers have attempted to answer the question of whether *Campylobacter* is a commensal organism or a pathogen of chickens [16, 17]. The answer to this question appears to depend on the genetics of the host and varies with infecting *Campylobacter* strain [18, 19]. However, whether these factors influence broiler chickens in commercial production has been challenged [20]. The outcomes of *Campylobacter* colonization of broiler chickens appear context specific, but in practice, any combination of microorganisms that produce conditions that modify the GI microbiota and reduce performance should be considered deleterious but do not necessarily constitute a disease [21–23].

Recent research has reported changes in the chicken microbiota in response to *Campylobacter* colonization [4, 24] with evidence of modification of the β-diversity of the cecal microbiota [25]. The objective of this study was to compare the microbiota of chickens that were not exposed to *Campylobacter*, with those exposed either at a young age (6 days of age) or at the age at which birds often become positive in commercial production (20 days of age), with a view to gain a better understanding of how the timing of *Campylobacter* colonization affects the microbiome and the innate and adaptive immune response.

## Methods

### Trial design

The first trial (referred to as trial L; late infection) monitored the development of the chicken gut microbiota and innate immune responses post-lag period colonization of broiler chickens by *Campylobacter jejuni* HPC5 [26, 27], at 20 days of age. Two groups of 35 birds were kept in pens until day 20 when trial L group 1 (TLG1) birds were administered with a placebo and trial L group 2 (TLG2) birds with *C*. *jejuni*, before being caged independently until the end of the study at day 35. Six birds from the TLG1 were euthanized for sampling at 22 days of age (da) and three at 28 and 35 da. Seven birds from the TLG2 group were euthanized for sampling at 22, 28, and 35 da. The second trial (referred to as trial E; early infection) monitored the development of the gut microbiota and innate immune responses of broiler chickens colonized early at 6 da by *C*. *jejuni*. Two groups of 35 birds were co-housed in pens until 6 da when trial E group 1 (TEG1) birds were administered with a placebo and trial E group 2 (TEG2) birds were administered with *C*. *jejuni*, before being caged independently until the end of the study at day 35. Seven birds from each group were euthanized for sampling at days 8, 15, 22, 28, and 35.

### Experimental animals

Day-of-hatch male Ross 308 broiler chicks were purchased from a local hatchery and brooded in floor pens on wood shavings until the day of *Campylobacter* colonization when they were randomly assigned on the basis of weight to one of two groups and held in two separate rooms under similar environmental conditions with category two biosecurity. Welfare monitoring of the chickens was undertaken either twice every 24 h or three times post *Campylobacter* colonization. Chickens had access to feed and water ad-libitum throughout the study. Chickens were fed on a wheat-based diet provided as a starter crumb 0–10 days, grower pellets 11–24 days, and finisher pellets 23–35 days. The starter diet contained wheat 59.9% (*w*/*w*), soybean meal 32.5% (*w*/*w*), soybean oil 3.65% (*w*/*w*), limestone 0.60%(*w*/*w*), calcium phosphate 1.59% (*w*/*w*), sodium bicarbonate 0.27% (*w*/*w*), salt 0.15% (*w*/*w*), lysine HCl 0.296% (*w*/*w*), DL-methionine 0.362% (*w*/*w*), threonine 0.134% (*w*/*w*), and the enzymes phytase and xylanase (dosed according to the instructions of the manufacturers DSM Nutritional Products Ltd. PO Box 2676 CH-4002 Basel, Switzerland). The grower and finisher diets increased the wheat content at the expense of soya meal by 2 and 5% respectively. The feed and paper liners on which the chicks were delivered were tested for *Salmonella* using standard enrichment procedures and found to be negative.

For TLG1, birds were administered a placebo of 1 ml of MRD (maximum recovery diluent; Oxoid, Basingstoke, UK) by oral gavage, and the TLG2 birds were administered 10^7^ CFU *C*. *jejuni* HPC5, a well-characterized broiler chicken isolate, in 1 ml MRD [26, 27]. TEG1 birds were administered with a placebo of MRD by oral gavage (0.1 ml) at 6 da birds and TEG2 with 10^7^ CFU *C*. *jejuni* strain HPC5 in 0.1 ml MRD. All feed consumed was recorded as were the body weights of the birds. Feed conversion ratios (FCR) were calculated as a ratio of feed consumed to the live weight of the birds.

Chickens were euthanized by either exposure to carbon dioxide gas or parenteral barbiturate overdose followed by cervical dislocation according to Schedule 1 of the UK Animals (Scientific Procedures) Act 1986. The birds were weighed before tissue and intestinal content samples were collected post-mortem. Ileal tissues were collected from approximately 3 cm distal to Meckel’s diverticulum and cecal tissues collected from the distal tips of the ceca. Intestinal tissues were immediately frozen in liquid nitrogen for subsequent RNA isolation or preserved in 10% (*w*/*v*) neutral buffered formalin (Fisher Scientific; Loughborough, UK) for histological assessment. Intestinal contents were collected and samples used either to acquire bacterial count data or for total genomic DNA extraction.

### Enumeration of bacteria from intestinal contents

Approximately 1 g of material was collected from both ceca and combined in pre-weighed universals before a 10% *w*/*v* suspension was prepared in MRD (Oxoid). *Campylobacter* were enumerated in triplicate from decimal dilutions prepared in MRD to 1 × 10^−7^ using a modification of the Miles and Misera technique. For each triplicate dilution set, five aliquots were dispensed onto CCDA agar (PO0119; Oxoid) prepared with the addition of agar to 2% (to prevent swarming) and with addition of CCDA Selective Supplement SR0155 (Oxoid). Plates were incubated at 42 °C in a microaerobic atmosphere (2% H_2_, 5% CO_2_, 5% O_2_, 88% N_2_) for 48 h (Don Whitley Scientific modified atmospheric cabinet, Shipley, UK). Coliforms were enumerated by application of aliquots of 100 μl from decimal dilutions of the cecal suspension to MacConkey No 3 agar (CM115; Oxoid) and incubation at 37 °C for 24 h. Lactic acid bacteria were enumerated by application of aliquots of 100 μl from decimal dilutions of the cecal suspension to MRS agar (CM0361; Oxoid) and incubation under anaerobic conditions at 30 °C for 48 h (Don Whitley Scientific anaerobic workstation). Between 30 and 300 colonies were counted on MacConkey No 3 and MRS agars, and the count per gram of cecal material was calculated by multiplying by the dilution factor.

### Histology

Samples of ileum for histological assessment were examined from each bird from both trials. The fixed tissue samples were dehydrated through a series of alcohol solutions, cleared in xylene, and finally embedded in paraffin wax (Microtechnical Services Ltd., Exeter, UK). Sections (3 to 5 μm thick) were prepared and stained with modified hematoxylin and eosin (H&E) using standard protocols. After staining, the slides were scanned by NanoZoomer Digital Pathology System (Hamamatsu, Welwyn Garden City, UK). Measurements of villus height and crypt depth were made using the NanoZoomer Digital Pathology Image Program (Hamamatsu) of 10 well-oriented villi scanned at × 40 magnification. The average of the 10 measurements was calculated per bird, from three or four birds per group, per time point. Villus height was measured from the tip of the villus to the crypt opening, and the associate crypt depth was measured from the base of the crypt to the level of the crypt opening. The ratio of villus height to relative crypt depth (V:C ratio) was calculated from these measurements. Heterophils were enumerated and any histopathological features recorded in a blind assessment of five random fields from each tissue section.

### RNA isolation and RT-qPCR of the cytokines and chemokines

RNA was isolated from cecal and ileal tissue biopsies using NucleoSpin RNA isolation kit (Macherey-Nagel, GmbH & co. KG, Düran DE) according to the manufacturer’s protocol with the following modifications. Tissue samples were homogenized in a lysis buffer with 2.8-mm ceramic beads (MO BIO Laboratories Inc., Carlsbad, USA) using TissueLyser II (Qiagen, Hilden, DE) prior to subsequent purification as described in the protocol. RNA was eluted in DEPC-treated water (Ambion ThermoFisher Scientific, UK) and stored at − 80 °C. RNA quality and concentration were assessed using Nanodrop ND-1000 Spectrophotometer (Labtech International Ltd., Uckfield, UK). The ratio 260/280 nm was in the range of 1.79 to 2.17 with the mean of 2.12 ± 0.01 for all RNA samples used.

Reverse transcription was performed with 1 μg of RNA using SuperScript II (Invitrogen Life Technologies, Carlsbad, USA) and random hexamers. Quantitative PCR reaction was performed with cDNA template derived from 4 ng of total RNA in triplicate using SYBR Green Master mix (Applied Biosystems, ThermoFisher Scientific). Cytokines and chemokines fold change were calculated using the comparative cycle threshold (Ct) method established by the manufacturer [28]. The average of the triplicate Ct values was used for analysis, and the target genes Ct values were normalized to those of the housekeeping gene encoding glyceraldehyde 3-phosphate dehydrogenase (GAPDH). Significance tests were calculated using ANOVA of the replicate the 2^−ΔCt^ values for each gene in the control and *Campylobacter*-colonized groups. The RNA levels of expression were determined by qPCR using the Roche Diagnostics LightCycler 480 (Hoffmann La Roche AG, CH). The primers used for qPCR of GAPDH, IFN-γ, IL-1β, IL-4, IL-6, IL-10, IL-17A, IL-17F, CXCLi1, and CXCLi2 [29–32] are presented in Table 1.

**Table 1:** Primer sequence 5′-3′ for the gene expression determined by qPCR

Target gene	Primer sequence (5′-3′)	Product size (bp)	NCBI accession number	Reference
GAPDH	F: GACGTGCAGCAGGAACACTA R: TCTCCATGGTGGTGA AGACA	343	NM_204305.1	[29]
IFN-γ	F: TGAGCCAGATTGTTTCGATG R: CTTGGCCAGGTCCATGATA	152	NM_205149.1	[29]
IL-1β	F: GGATTCTGAGCACACCACAGT R: TCTGGTTGATGTCGAAGATGTC	272	NM_204524.1	[29]
IL-4	F: GGAGAGCATCCGGATAGTGA R: TGACGCATGTTGAGGAAGAG	186	NM_001007079.1	[29]
IL-10	F: GCTGCGCTTCTACACAGATG R: TCCCGTTCTCATCCATCTTC	203	NM_001004414.2	[29]
IL-6	F: GCTCGCCGGCTTCGA R: GGTAGGTCTGAAAGGCGAACAG	71	NM_204628.1	[30]
IL17-A	F: CATGGGATTACAGGATCGATGA R: GCGGCACTGGGCATCA	68	NM_204460.1	[31]
IL17-F	F: TGACCCTGCCTCTAGGATGATC R: GGGTCCTCATCGAGCCTGTA	78	XM_426223.5	[31]
ChCXCLi1	F: CCGATGCCAGTGCATAGAG R: CCTTGTCCAGAATTGCCTTG	191	NM_205018.1	[32]
ChCXCLi2	F: CCTGGTTTCAGCTGCTCTGT R: GCGTCAGCTTCACATCTTGA	128	NM_205498.1	[32]

### DNA extraction and PCR amplification of 16S rRNA gene sequences and microbiota diversity analysis

Bacterial DNA was isolated from 0.25 g cecal content using the PowerSoil DNA Isolation Kit (MO Bio Laboratories) according to the manufacturer’s instructions. Using the isolated DNA as a template, the V4 region of the bacterial 16S rRNA gene was PCR amplified using primers 515f (5′ GTGCCAGCMGCCGCGGTAA 3′) and 806r (5′ GGACTACHVGGGTWTCTAAT 3′) [33]. Amplicons were then sequenced on the Illumina MiSeq platform using 2 × 250 bp cycles. These sequence data are deposited in the NCBI database within the Bioproject PRJNA380214 under the SRA study SRP133552.

Prior to metagenomic analysis, sequence reads with a quality score mean below 30 were removed using Prinseq [34]. The 16S rRNA sequence analysis was performed using Mothur v. 1.39 [35]. Analysis was performed as according to the MiSeq SOP (accessed online 28/06/2017; [36]). The 16S rRNA gene sequences were aligned against a reference alignment based on the SILVA rRNA database [37] for use in Mothur (available at: https://www.mothur.org/wiki/Silva_reference_files), and clustered into operational taxonomic units (OTUs) using the opticlust clustering algorithm [38]. The nearest 16S rRNA gene sequence identities to the OTUs are reported on the basis of BLASTn searches if data matches are from type cultures with a BLAST identity ≥ 99%. If not, the consensus taxonomy of the OTUs is reported as generated using the classify.otu command in Mothur with reference data from the Ribosomal Database Project (version 14) [39, 40] adapted for use in Mothur (available at: https://www.mothur.org/wiki/RDP_reference_files).

### Data and statistical analysis

For the microbiota beta diversity analysis, Bray-Curtis distances were tested for significance using analysis of molecular variance (AMOVA) implemented within Mothur [38]. For alpha diversity, inverse Simpsons indices and species abundance were tested using Kruskal-Wallis test followed by Dunn’s multiple comparison test with Benjamini-Hochberg *p* value correction within R [41, 42] using Dunn.test 1.3.4 package [43]. The Shapiro-Wilk normality test for data distribution analysis was used from within GraphPad Prism version 7.00 for Windows (GraphPad Software, La Jolla, USA, http://www.graphpad.com). Data processing and ordination were performed using R project. Statistical differences between *Campylobacter* and non-*Campylobacter*-colonized groups with respect to the zootechnical parameters were determined using repeated measures ANOVA implemented in Genstat release 19.1 (VSN International, UK). *Campylobacter* viable counts exhibiting a normal distribution, heterophil counts, and the villus and crypt measurements were made using single-factor ANOVA with < 0.05 used as the level significance. For microbiota data sets, non-parametric Mann-Whitney tests were performed. Linear discriminant analysis effect size (LEfSe) was used to identify differentially abundant OTUs (available at https://bitbucket.org/nsegata/lefse/overview) using a minimum cutoff of 0.05% [44]. Analysis of similarity (ANOSIM) with the Benjamini-Hochberg correction for multiple comparisons with analysis of similarity percentages (SIMPER) [45] was used to determine the contribution of each taxonomic unit to the Bray-Curtis dissimilarity of pairs of distinct sample groups using the vegan package [46] in R using a script by Andrew Steinberger (https://github.com/asteinberger9/seq_scripts) as previously reported for the interrogation of 16S rDNA OTUs [47].

## Results

### Growth rate and feed conversion ratio (FCR) of birds infected with *C*. *jejuni* HPC5

Each bird was weighed regularly throughout the experimental period to compare the growth of birds infected with *C*. *jejuni* HPC5 and uninfected control birds. There were significant differences between the weights of the control and experimental birds infected at 6 da (TEG; *p* < 0.01). Notably, these differences were evident at 2 and 9 days post-infection (dpi), when the control birds in TEG1 were significantly heavier (*p* < 0.01) than infected birds (TEG2). The reduced weights of the TEG2 birds at 2 dpi coincided with the observation of temporary diarrhea that resolved within 72 h. However, by the end of the rearing cycle (35 da), there was no significant difference (*p* > 0.05) in the weights of the birds infected with *Campylobacter* compared to uninfected controls (Additional file 1). In contrast, the weights of birds in TLG1 were not significantly different to those in TLG2. The cumulative FCR up to 35 days for TLG1 (*n* = 8) and TLG2 (*n* = 7) were 1.52 and 1.56 respectively while the FCR for TEG1 (*n* = 10) and TEG2 (*n* = 7) were 1.48 and 1.45 respectively. Breed performance targets for commercial broiler chickens suggest an FCR of 1.54 at 35 da.

### *Campylobacter jejuni* colonization

**Fig. 1:**
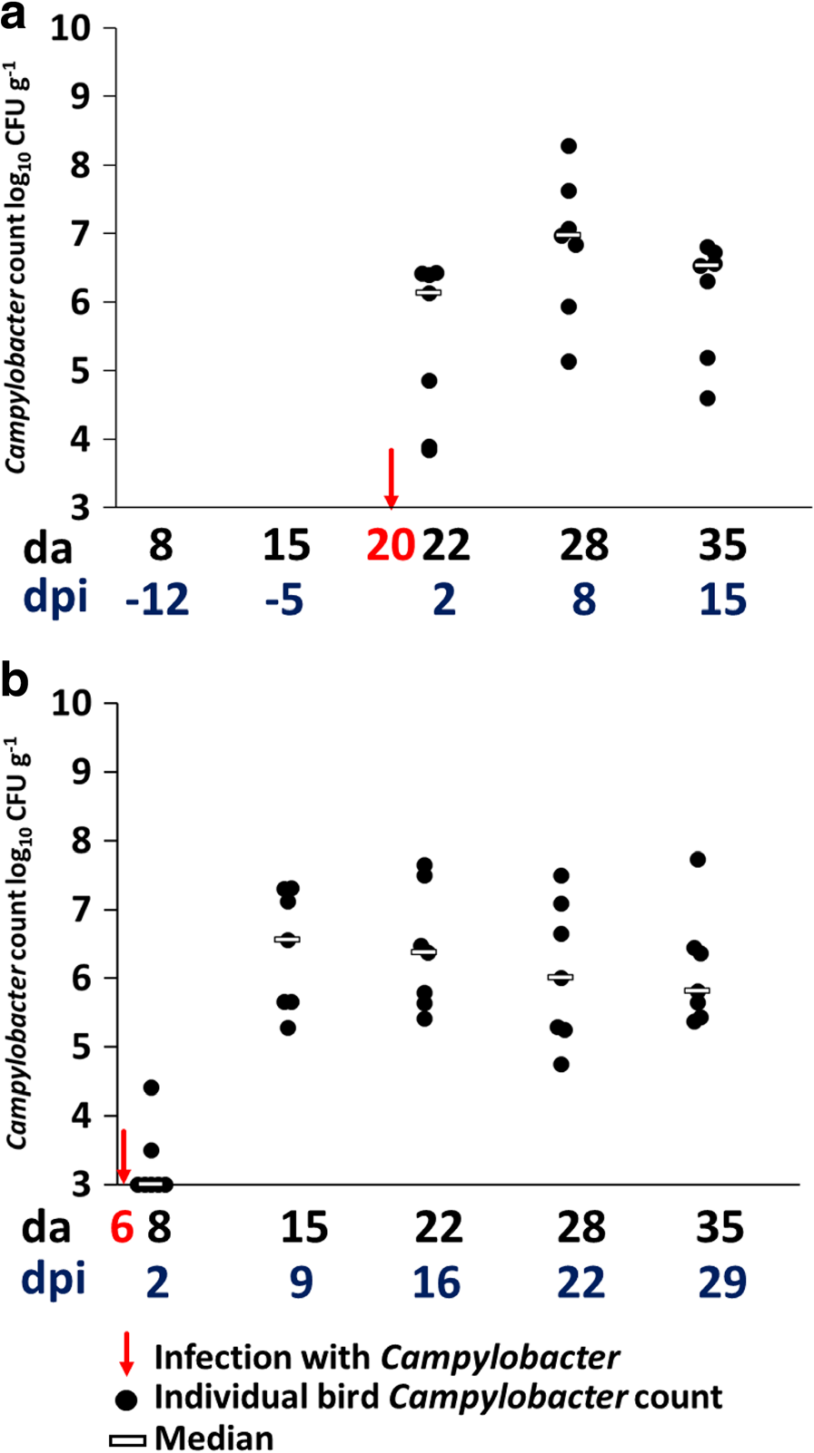
Viable counts of *Campylobacter* colonization of the cecal lumen. **a** TLG2 birds infected at 20 da with no significant differences in the counts post infection. **b** TEG2 birds infected at 6 da. No significant differences in the counts from 9 days post infection were observed (*p* > 0.05; ANOVA)

All birds were culture negative for *Campylobacter* spp. until experimental infection with *C*. *jejuni* and control birds remained culture negative for *Campylobacter* spp. throughout the study. *Campylobacter* viable counts of the cecal contents recovered at the end of the rearing cycle were high independent of age at infection (mean *Campylobacter* density = 6.1 log_10_ CFU g^−1^; Fig. 1a, b). The dynamics of colonization were however affected by the age at which birds were infected with *Campylobacter*. Birds from TLG2, infected at 20 da all exhibited cecal colonization with *C*. *jejuni* (mean 5.1 log_10_ CFU g^−1^) at 2 dpi, with all the birds sampled at each time point thereafter (*n* = 7) showing colonization until the end of the rearing cycle at 35 da (15 dpi; Fig. 1a). Only two of seven birds sampled from TEG2 at 2 dpi had levels of *Campylobacter* in their ceca above the limit of detection, but by the next sample point at 9 dpi, all birds showed colonization to levels that remained similar after this stage (*p* > 0.05; Fig. 1b).

### Colonization with C. *jejuni* affects intestinal villus and crypt metrics

**Fig. 2:**
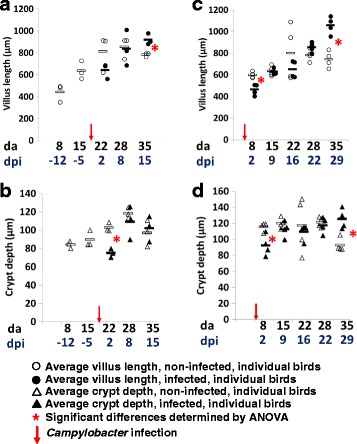
Comparison of the average villus/crypt measurements from H&E-stained sections of ileum. **a** TLG1 and TLG2 villus length. **b** TLG1 and TLG2 crypt depth. **c** TEG1 and TEG2 villus length. **d** TEG1 and TEG2 crypt depth length. Measurements were taken from 10 villi or 10 crypts per bird. Three or four birds were sampled from each group for each time point

Heterophil infiltration counts were determined in a blind assessment of formalin-fixed H&E-stained ileum sections (Additional file 2 contains typical examples) to reveal significant differences using ANOVA at 2 (*p* = 0.02) and 9 dpi (*p* = 0.01) for birds infected with *C*. *jejuni* at 6 da (TEG2) compared to uninfected birds but were not significant thereafter (*p* > 0.05). Heterophil infiltration at 2 and 9 dpi was accompanied by mild multi-focal villous blunting, with evidence of mild edema and villous fusion. For the birds infected at 20 da, significant increases in the heterophil counts were observed in the ileum sections of the infected birds (TLG2) at 2 (*p* = 0.04) and 8 dpi (*p* = 0.01). However, villus crypt ratios obtained from measurements taken from H&E-stained sections of the ileum, comparing uninfected TLG1 to infected TLG2 from 3 to 4 birds from each group, at each sample time point, revealed no significant difference (*p* > 0.05) between the two groups at any age. The same comparison, made with H&E-stained sections of the ileum from uninfected TEG1 and infected TEG2, showed no significant difference between uninfected and infected birds. However, when comparing the villus height and the crypt depth measurements separately, significant differences using ANOVA were noted between the infected and uninfected birds (Fig. 2). Villus length and crypt depth were reduced immediately after infection but both measurements were increased at the end of the rearing period. TLG2 birds show a significant reduction (*p* = 0.0005) in crypt depth, 2 dpi, combined with an observable, but not statistically significant (*p* = 0.13), reduction in the villus height compared to uninfected TLG1 birds. The measurements of the villi and crypts of birds in TLG1 and TLG2 were similar (*p* > 0.05) at 8 dpi, but at the final sampling point (15 dpi), the villus height from the *Campylobacter*-infected TLG2 birds was increased compared to the uninfected TLG1 birds at the same age (*p* = 0.0004) although the crypt depths were not significantly different (*p* = 0.7). The birds in TEG2 showed a similar pattern. Immediately following infection (2 dpi), the villi were significantly reduced in height (*p* = 0.003) and the crypts reduced in depth (*p* = 0.02) compared to the control birds (TEG1). However, by the next sample point (9 dpi), there was no significant difference in villus height or in the crypt depth for birds in TEG1 compared to TEG2 (*p* > 0.05). No significant differences were observed thereafter until the final sample point (29 dpi), where the villi were significantly longer (*p* = 0.004) and the crypts significantly deeper (*p* = 0.008) in the infected TEG2 birds compared to the uninfected TEG1 birds.

### Effect of *C*. *jejuni* colonization on cytokine and chemokine gene expression

**Fig. 3:**
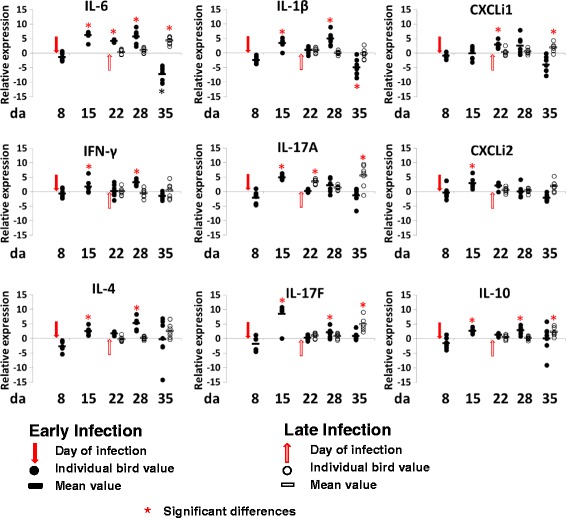
Relative change in expression of cytokines and chemokines in ileum tissues. Relative gene expression represents log_2_ ratio infected/non-infected from qPCR of infected birds (TLG2 and TEG2) compared to expression in tissues from non-infected birds (TLG1 and TEG1). Significant differences between 2^-ΔCt^ values of the control and *Campylobacter*-colonized groups are indicated by an asterisk (ANOVA *p* < 0.05, *) for the expression of each gene at the corresponding time points

**Fig. 4:**
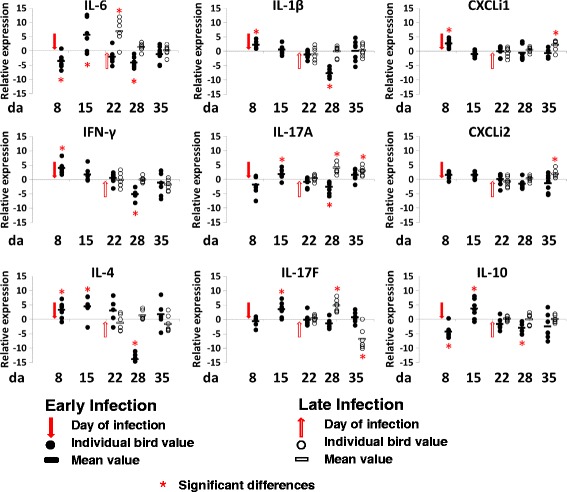
Relative change in expression of cytokines and chemokines in cecal tissues. Relative gene expression represents log_2_ ratio infected/non-infected from qPCR of infected birds (TLG2 and TEG2) compared to expression in tissues from non-infected birds (TLG1 and TEG1). Significant differences between 2^−ΔCt^ values of the control and *Campylobacter* colonized groups are indicated by an asterisk (ANOVA *p* < 0.05, *) for the expression of each gene at the corresponding time points

The inflammatory effect of *C*. *jejuni* colonization was assessed by quantification of the relative expression of cytokines and chemokine gene transcripts in ileal and cecal tissue biopsies (Figs. 3 and 4) representing major inflammatory pathways in chickens [30]. The cytokines IL-17F, IL-17A, IL-6, and IL-1β and chemokines CXCLi1 and CXCLi2, also known as ChIL-8, have previously been described as markers of the Th17 pathway. Whereas IFN-γ is related to the Th1 pathway, IL-4 is connected to the Th2 pathway and IL-10 is produced by regulatory T cells (Treg) to control the inflammatory effects of the Th cell responses. There was no significant change in the cytokine and chemokine expression in ileum tissues (Fig. 3) at 2 dpi following infection at 6 da in TEG2 birds. However, at 9 dpi, most cytokines showed a significant (*p* < 0.05) increase in expression compared to controls corresponding to the increasing levels of colonization observed in Fig. 1b. Notably, increases in IFN-γ, IL-4, and IL-17A provided evidence for activation of Th1, Th2, and Th17 pathways but these were also accompanied by an increase in IL-10. Levels of expression remained higher than controls for the majority of the *Campylobacter*-colonized birds until 29 dpi when they were reduced to similar or lower levels than control birds. Changes in cytokine expression in response to infection by *Campylobacter* at 20 da in TLG2 birds was characterized in the ileum tissues (Fig. 3) by a significant (*p* < 0.05) increase in most of the cytokine expression at 15 dpi compared to uninfected TLG1 birds, with the exception of IFN-γ and IL-1β. Prior to that time point, the level of cytokine expression was not significantly different to the non-infected birds (TLG1) at 2 dpi and 8 dpi despite a high level of *Campylobacter* colonization detected as early as 2 dpi, although the cytokine IL-17A showed a significant increase in expression from 2 dpi onwards in the TLG2 birds. Interestingly, most of the immune response markers were upregulated at an earlier stage during the infection in TEG2 birds (at 9 dpi) rather than in TLG2 birds (at 15 dpi) despite the high level of *Campylobacter* detected at 2 dpi in the TLG2 birds.

Changes in cytokine and chemokine expression in cecal tissues in response to colonization by *Campylobacter* (Fig. 4) at 6 da were characterized by significant increases in IFN-γ, IL-1β, IL-4, and CXCLi1 and a decrease in IL-6 and IL-10 at 2 dpi in TEG2 birds. A week later at 9 dpi, the expression of IL-6 was increased along with IL-17A, IL-17F, IL-10, and IL-4. By 16 dpi, their level of expression was not significantly different to the uninfected TEG1 birds, and at 22 dpi, the majority of the cytokines showed a significant (*p* < 0.05) reduction in expression compared to control birds TEG1, with the exception of IL-17F and CXCLi1. Finally, at the last time point 29 dpi, the cytokine and chemokine levels had recovered to levels not significantly different to the non-infected control (TEG1). Cecal tissues of birds infected at 20 da did not show the concerted Th1 and Th2 immune responses relative to the non-infected control birds at 2 dpi that the birds colonized at 6 da experienced. However, IL-6 showed a significant increase of 35-fold, followed by increases in the levels of IL-17A and IL-17F at 8 dpi and ultimately increased CXCLi1, CXCLi2, and IL-17A at 15 dpi. Following infection with *Campylobacter*, the immune response in the cecal tissues appears to be more focused on the Th17 pathway featuring IL-6 induction with IL-17A and IL-17F responses, as compared to that observed in the ileum tissues.

### Effect of *C*. *jejuni* colonization on the microbiota of the cecal lumen

DNA sequencing of the V4 regions of 16S rRNA genes was used to estimate the diversity and abundance of the cecal luminal microbiota of birds from the TEG and TLG experiments. A total of 6,947,272 quality-controlled sequence reads from 107 samples were resolved in to 7646 OTUs (distance 0.03) that fall into 23 phyla. As described previously for chicken cecal microbiota, *Firmicutes* dominate with a mean abundance of 87.57% (83.89–93.91%) over all samples from 8 days of age onwards and followed by *Proteobacteria* at 6.43% (3.47–8.77%) [8, 48]. The relative abundances of these phyla for all samples are presented in Additional file 3. The sequence reads were subsampled at 16,319 reads per sample for subsequent analysis.

**Fig. 5:**
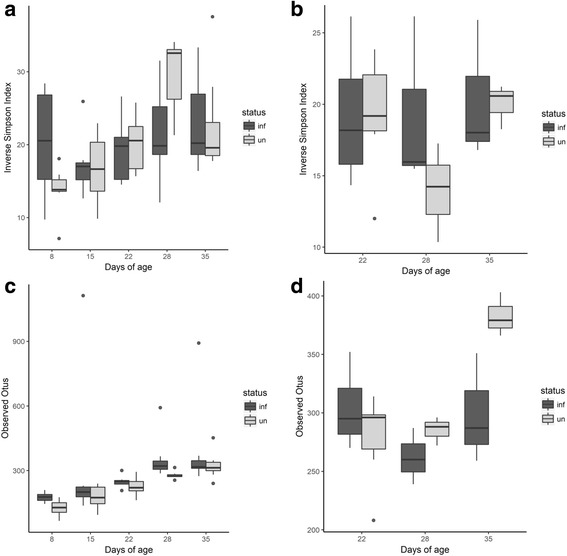
Estimates of α-diversity and richness for TEG and TLG microbial communities from cecal contents. Inverse Simpsons indices (**a**, **b**) and species richness estimates (**c**, **d**) from 16,319 subsampled sequences for the non-colonized control (gray bars) and *C*. *jejuni*-colonized (dark gray bars) chickens. The charts are presented with respect to the age of the birds colonized at either 6 days (TEG2) or 20 days (TLG2). **a** Inverse Simpsons index of TEG1 and TEG2. **b** Inverse Simpsons index of TLG1 and TLG2. **c** OTU counts of TEG1 and TEG2. **d** OTU counts from TLG1 and TLG2. Data are presented as a box and whisker plot (in the style of Tukey). The solid black line indicates the median, and the top and bottom of the shaded boxes indicate the 25th and 75th percentiles. Whiskers indicate maximum and minimum values, unless these values exceed 1.5-fold the interquartile range. Outlying data are plotted as individual markers

Figure 5a, b shows estimates of the diversity of the microbiota, presented as plots of the inverse Simpsons measure of α-diversity. The α-diversity of the cecal microbiotas from birds of TEG or TLG was not affected by *C*. *jejuni* colonization (*p* > 0.05). However, an age-linked increase in alpha diversity was evident for the non-colonized TEG birds between days 8 and 28 (*p* = 0.0005). Figure 5c, d shows that richness of the cecal microbial communities are generally not affected by *C*. *jejuni* colonization (*p* > 0.05) with the exception of a significant increase in the observed OTUs at day 28 for the *C*. *jejuni*-colonized TEG birds.

Bray-Curtis indices of dissimilarity demonstrate differences in species composition between communities on the basis of age and *C*. *jejuni* infection status. The cecal microbiota of birds infected with *Campylobacter* at 6 da (TEG2) was significantly different to age-matched controls at 2, 16, and 22 dpi (*p* < 0.05, AMOVA). Principal component analysis of these data demonstrates clustering of the data with respect to age (Additional file 4). The exception to this is the data at 15 da (9 dpi), which exhibit similarities with either the pre- or proceeding data. The transition in the microbiota at 15 da is also marked in the microbial counts obtained for coliforms and lactic acid bacteria by a shift in the dominance of the coliform count to that of lactic acid bacteria after the 15 da time point independent of the *C*. *jejuni* colonization status (Additional file 5). Bray-Curtis indices indicate the microbiota of birds exposed to *Campylobacter* at 20 da (TLG2) was significantly different from uninfected birds immediately post-infection (2 dpi; *p* < 0.001, AMOVA), but could not be distinguished from controls at subsequent stages of the rearing cycle (*p* > 0.05, AMOVA).

**Fig. 6:**
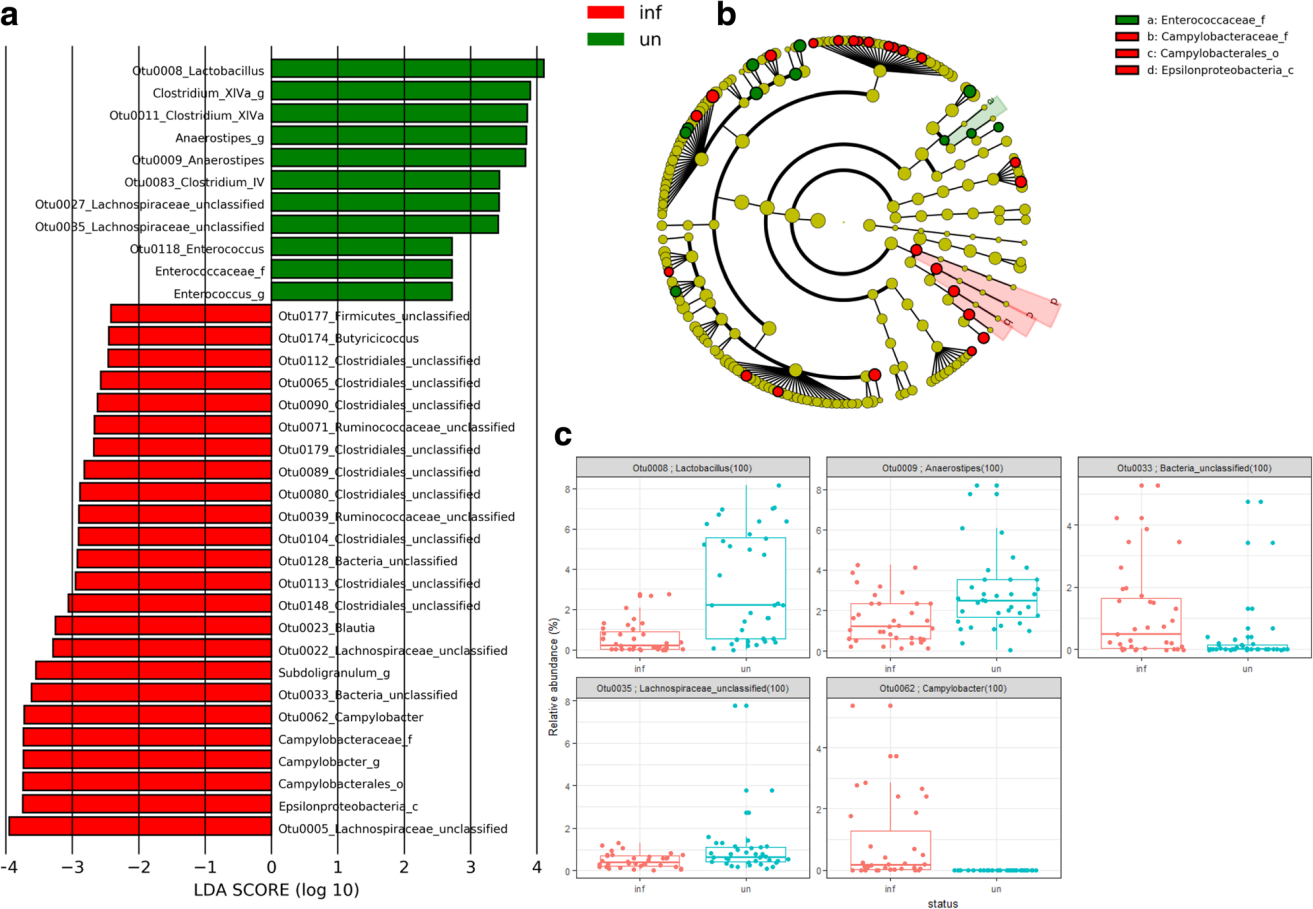
Differential abundance of members of the cecal microbial communities of TEG *C*. *jejuni*-colonized and non-colonized broiler chickens. **a** Histogram of the LDA scores computed for features differentially abundant between *C*. *jejuni*-colonized broiler chickens from 6 da (denoted as “inf” by red bars) and non-colonized birds (denoted as “un” by green bars). LEfSe identifies which clades amongst those detected as statistically differential will explain the greatest differences between the communities [42]. **b** A taxonomic representation of the clades responsible for the greatest differences based on the Ribosomal Database Project [39], where red circles represent those of greater abundance in the *C*. *jejuni*-colonized birds, green circles for those of non-colonized birds, and yellow for non-significant differences. The diameters of the circles are proportional to the taxon’s abundance. The representation highlights the presence of the differentially abundant taxanomic levels containing *Campylobacter* (_f family, _o order and _c class) as concentric arcs labeled a to c. **c** Plots of the relative abundance differences between *C*. *jejuni*-colonized (denoted as inf in red) and non-colonized chickens (denoted as un in blue) for TEG when calculated using ANOSIM from Bray-Curtis indices and identified by SIMPER. Each data point represents the relative OTU abundance in a single bird. The horizontal line indicates the median, and the top and bottom of the boxes indicate the 25th and 75th percentiles. Whiskers indicate maximum and minimum values with the exception of those exceeding 1.5-fold the interquartile range

Linear discriminant analysis effect size (LEfSe) was applied to identify differentially abundant OTUs between *Campylobacter*-infected and non-infected birds. Figure 6a shows the significant differentially abundant OTUs for the entire TEG microbiota that include the colonizing *C*. *jejuni* HPC5 (OTU0062) at all taxonomic levels as indicated in Fig. 6b. Only those microorganisms that are noted as type cultures and had BLASTn identities ≥ 99% are reported to species level; otherwise, the consensus taxonomies with the corresponding OTU numbers are reported. Differential abundance of members the dominant *Firmicutes* phylum was evident in response to *C*. *jejuni* colonization. *C*. *jejuni-*colonized birds exhibited increased abundance of *Lachnospiraceae* ssp. OTU0005 and OTU0022, *Blautia* ssp. OTU0023, *Ruminococcaceae* OTU0039 and OTU0071 in addition to several unclassified members of the *Clostridiales* class. In the non-colonized birds, LEfSe highlights the greater differential abundance of *Lactobacillus* OTU0008, *Anaerostipes butyraticus* OTU0009, *Clostridium* XIVa OTU0011, *Lachnospiraceae* spp. OTU0035 and OTU0027, *Clostridium* IV OTU0083, and *Enterococcus* OTU0118. The differential abundances identified by LEfSe for age-matched colonized and non-colonized birds are presented in Additional file 6. At 8 da (2 dpi), the corresponding *C*. *jejuni* OTU was not significantly more abundant using the 0.05% cutoff adopted for all samples, although it should be noted that viable *C*. *jejuni* were only detected by culture in the ceca of 2 of 7 birds from the TEG2 group at this early time point. As an alternative approach, the OTUs contributing to differences in the Bray-Curtis dissimilarity indices were identified by analysis of similarity percentages (SIMPER). Figure 6c shows box-whisker plots of the relative OTU abundances between *C*. *jejuni*-colonized and non-colonized birds for five OTUs identified using SIMPER (*p* adj < 0.05). All five OTUs coincide with those identified as differentially abundant by LEfSe.

**Fig. 7:**
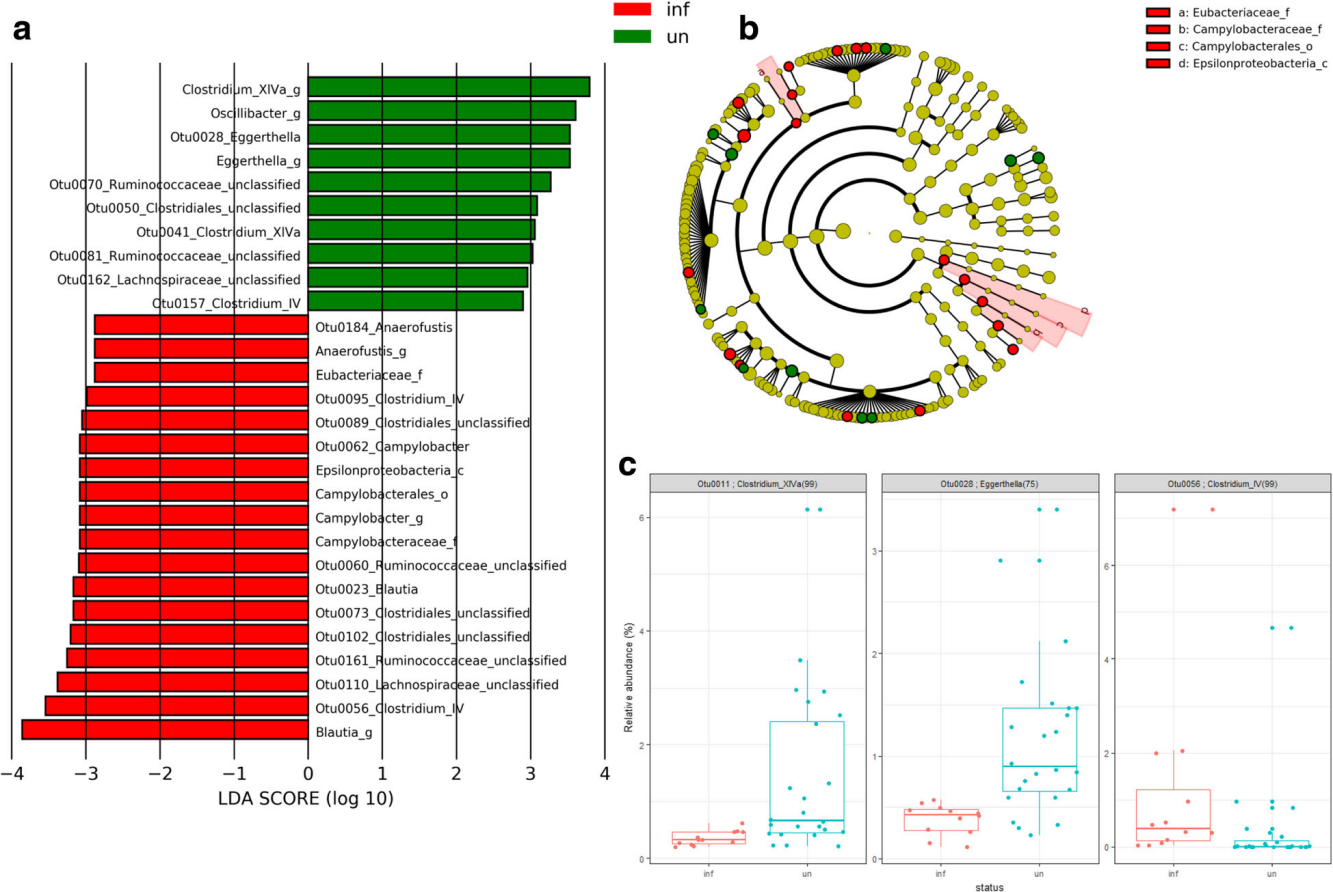
Differential abundance of members of the cecal microbial communities of TLG *C*. *jejuni*-colonized and non-colonized broiler chickens. **a** Histogram of the LDA scores computed for features differentially abundant between *C*. *jejuni*-colonized broiler chickens from 20 da (denoted as “inf” by red bars) and non-colonized birds (denoted as “un” by green bars). LEfSe identifies which clades amongst those detected as statistically differential will explain the greatest differences between the communities [42]. **b** A taxonomic representation of the clades responsible for the greatest differences based on the Ribosomal Database Project [39], where red circles represent those of greater abundance in the *C*. *jejuni*-colonized birds, green circles for those of non-colonized birds, and yellow for non-significant differences. The diameters of the circles are proportional to the taxon’s abundance. The representation highlights the presence of the differentially abundant taxanomic levels containing *Campylobacter* (_f family, _o order and _c class) as concentric arcs labeled a to c. **c** Plots of the relative abundance differences between *C*. *jejuni*-colonized (denoted as inf in red) and non-colonized chickens (denoted as un in blue) for TEG when calculated using ANOSIM from Bray-Curtis indices and identified by SIMPER. Each data point represents the relative OTU abundance in a single bird. The horizontal line indicates the median, and the top and bottom of the boxes indicate the 25th and 75th percentiles. Whiskers indicate maximum and minimum values with the exception of those exceeding 1.5-fold the interquartile range

LEfSe analysis of the TLG differentially abundant OTUs between *C. jejuni*-colonized and non-colonized birds are presented in Fig. 7a with the corresponding phylogenetic relationships in Fig. 7b. Notably, three of the OTUs identified with increased abundance in the *C*. *jejuni*-colonized TLG birds coincided with those from the TEG comparison: *Lachnospiraceae* ssp. OTU0022, *Blautia* ssp. OTU0023, and unclassified *Clostridiales* OTU0089. In the non-colonized birds, LEfSe identified greater differential abundance of *Eggerthella* OTU0028, *Clostridium* XIVa OTU0041, unclassified *Clostridiales* OTU0050, *Ruminococcaceae* OTU0070 and OTU0081, and *Lachnospiraceae* spp. OTU0162. Figure 7c shows box-whisker plots of the relative OTU abundances between *C*. *jejuni*-colonized and non-colonized birds for three OTUs identified using SIMPER (*p* adj < 0.05). The increased abundances corresponding to *Eggerthella* OTU0028 in the colonized birds and *Clostridium* IV OTU0056 in the non-colonized birds also feature in those identified as those responsible for the differential abundance by LEfSe for TLG. The taxon *Clostridium* XIVa (OTU0011 and OTU0041) shows differential increases in abundance in the non-colonized birds that contributes to the dissimilarity between the *C*. *jejuni*-colonized and non-colonized groups for TEG and TLG.

## Discussion

Recent reports have linked *Campylobacter* colonization of broiler chickens with reduced economic performance in terms of an increase in cumulative FCR. Evidence for this comes from correlating poor economically performing farms with high *Campylobacter* prevalence [49] and from smaller scale experimental trials [50]. There were distinct differences in zootechnical performance between the two independent trials reported here despite similar diets and controlled housing, but these were independent of *Campylobacter* colonization. The TLG trial showed FCRs of 1.52 and 1.56 respectively for TLG1 and TLG2, whereas the TEG trial had FCRs of 1.48 and 1.45 respectively for TEG1 and TEG2. The between trial differences could not be explained by either an increase in the α-diversity or richness of the cecal microbiota.

Early infection of the birds in TEG2 resulted in significantly reduced live weights compared to control birds at 2 and 9 dpi, but this appeared to be a temporary set-back that the birds recovered from, as there were no significant differences between infected and non-infected bird weights thereafter. There were no significant difference (*p* > 0.05) between non-infected TLG1 and infected TLG2 bird weights. Within trial performance differences between the infected and non-infected birds within the current study were marginal considering the limited number of birds but appear to be associated with differences in feed intake post *C*. *jejuni* colonization. Chickens were housed under favorable conditions in this study, so how these observations may play out in commercial settings with greater stocking densities and environmental challenge requires consideration. Studies of natural infection reported by Gormley et al. found no correlation between bird body weights and cecal loads at slaughter age [20]. *C*. *jejuni* have been reported to exhibit strain-dependent differences in the outcomes of infection [51], which could contribute to differences in *Campylobacter*-positive flock performances. Exposure to multiple *Campylobacter* strains that result in succession of the fittest is indicative of multi-factorial challenges in barn-reared birds [52], which are likely to influence flock performance and associated negative welfare indicators.

*Campylobacter jejuni* colonization to high levels occurred more rapidly in birds infected at the end of the lag phase (20 da; TLG2) than in birds infected at 6 da (TEG2), which exhibited low or undetectable levels of cecal colonization at 2 dpi but reached full colonization at 9 dpi. The reduced weight gain and changes in villus/crypt measurements observed were more evident in the TEG2 birds at 2 dpi than at later sample points when levels of colonization were higher, suggesting that the level of *Campylobacter* colonization was not necessarily linked to these responses. The fact that similar responses were present in all the birds despite the majority being culture negative for *C*. *jejuni* suggests that following administration of the bacteria, the organism is able to persist, affect shifts in the microbial community, and affect physiological change, but not necessarily multiply to the extent that it can be detected by culture from cecal content. Clearly, birds at 6 da exhibit colonization resistance, which may in part be due to the presence of maternal antibodies [12, 13] that act to prevent immediate high-level colonization but are absent by 20 da. Regarding the lag phase observed in commercial production whereby flocks remain *Campylobacter* negative until the birds are 2 weeks of age, the current study indicates that chickens can become infected at any time during the rearing period but the colonizing campylobacters only multiply to the extent of being detectable and efficiently transmittable when the birds are over 2 weeks old, which lends support of the proposed mechanism of age-dependent transmission [15].

A healthy well-differentiated intestinal mucosa consists of long regular villi with high villus/crypt ratios [53]. Awad et al. [50] reported that Ross 308 birds infected with *Campylobacter* at 14 days of age (approximately half way between the two infection points described here) were found to have decreased villus height, crypt depth, and villus surface area by 21 days of age and were accompanied by changes in ion transport and barrier function compared to controls. Birds from TEG2 similarly showed a reduction in villus height and crypt depth compared to TEG1, immediately following infection (2 dpi) but by 9 dpi there was no significant difference, and by 29 dpi, the *Campylobacter* infected TEG2 birds actually had longer villi and deeper crypts than TEG1. This pattern would indicate that infection with *Campylobacter* can result in rapid changes in villus length, which can be correlated with temporary reduced weight gain and diarrhea, perhaps due to reduced nutrient absorption. However, this was followed by a fairly rapid recovery, within 9 days and in the long term, increased villus length compared to non-infected controls. Later infection with *Campylobacter* had a significant, but less drastic effect on villus heights immediately following infection of TLG2 compared to TLG1 uninfected birds. This was followed by a rapid recovery and by the end of rearing period exhibited increased villus height and depth compared to uninfected controls, similar to the observations made for TEG2.

Infection of the gastrointestinal tract by pathogens is detected by the host immune system which then responds via a complex interconnecting system of pathways involving the innate and adaptive immune systems. Cytokines play an important part in intracellular and extracellular immunity against pathogens and also in regulating the response appropriately. In chickens, the effector T cell pathway Th17 includes IL-17A and IL-17F and is thought to be important in limiting both invasion and colonization of bacterial pathogens in the gastrointestinal tract that include *Campylobacter* [31]. Cytokine expression in response to infection by *C*. *jejuni* in chickens challenged at 20 da, in TLG2, confirmed the upregulation of IL-6, IL-17A, and IL-17F (*p* < 0.01) reported by Reid et al. [31], although prolonged diarrhea was not observed as reported for faster growing broiler chicken breeds [19]. All TLG2 birds showed cecal colonization with *C*. *jejuni* at 2 dpi (mean 5.1 log_10_ CFU g^−1^) that was accompanied by an increase in IL-6 expression. For birds infected at 6 da, the kinetics of the response was different with no increase in IL-6 expression and largely undetectable levels of cecal *C*. *jejuni* colonization at 2 dpi. Instead a relative increase in IFN-γ and IL-4 were observed (*p* < 0.05), characteristic of Th1 and Th2 pathways. However, by 9 dpi, colonization of all birds was evident (mean 6.1 log_10_ CFU g^−1^), which coincided with increased expression of IL-6, IL-17A, and IL-17F (*p* < 0.01). At 9 dpi, IL-10 expression was also notably upregulated in ileal and cecal tissues (*p* < 0.05), which may account for the subsequent suppression of the pro-inflammatory cytokines, and in particular the declines in IL-6, IL-17A, and IL-17F. Cytokine IL-10 is produced by regulatory T (Treg) cells to control Th cell pro-inflammatory responses and prevent damage to affected tissues. The differential expression of IL-10 in broiler chicken breeds has been reported to be critical to the outcome of *C*. *jejuni* infection in terms of inflammation and diarrhea [19]. In this context, birds infected at 20 da did not show a significant increase in IL-10 in the ceca but a response was evident in the ileum by 35 da. These tissues exhibited increased levels of IL-17A until the end of the rearing period at 35 da. *C*. *jejuni* generally colonize the ceca of chickens to far greater cell densities; it is therefore of interest that the chickens did not upregulate IL-10 in their ceca within the 35 da rearing period that is typical of commercial flocks. A differential effect on the persistence of the pro-inflammatory response to *Campylobacter* colonization of a popular broiler chicken breed depending on the age of the bird is of significance to the poultry industry. Late colonized birds will be subject to an on-going pro-inflammatory response, the outcome of which will likely depend on the resident intestinal microbiota.

AMOVA of Bray-Curtis indices indicate significant differences between the cecal microbiota compositions of control birds and the TEG2 group colonized with *C*. *jejuni* at 2, 16, and 22 dpi (*p* < 0.05). Inspection of the PCoA plots shows partition of the control bird indices at 15 and 22 da as the microbiota undergoes a transition from a juvenile to a more mature composition (Additional file 4). The timing of the shift in microbiota does not correspond with any of the programmed changes in diet. The *C*. *jejuni*-colonized birds also exhibit the transition at 15 da but show less variance at 22 da. The transition is also marked in the ratio of coliforms to lactic acid bacteria counts by a shift in the dominance from coliform to lactic acid bacteria after the time point independent of whether or not the birds were colonized by *C*. *jejuni* (Additional file 5). Any differences between the *Campylobacter*-colonized and control groups will be superimposed upon this developmental transition. Han et al. [22] examined the influence of *C*. *jejuni* infection with age by inoculating broiler chickens with log_10_ 4 CFU *C*. *jejuni* at 1, 10, 22, and 31 da and determining the colonization levels and immune functions in the colonized birds. Circulating *C*. *jejuni*-specific maternal antibodies were detected in control birds from 3 da but absent by 15 da, which correlates well with the transition in microbiota we observe at that time point. A recent study by Ballou et al. [8] examined the development of the layer chicken microbiome and the effect of microbial interventions in the form of administering microbial treatments of probiotic bacteria and live *Salmonella* vaccines. These authors demonstrate changes in the microbiota with treatment and suggest that the functional impact of these treatments can stimulate greater differences at 14 da rather than later. Similarly, Awad et al. [54] recently noted a transition in the cecal microbiota of broiler chickens post 14 da with a relative increase in *Firmicutes* and *Tenericutes* at the expense of *Proteobacteria*. These authors also reported changes in the abundance of the microbial communities in response to *C*. *jejuni* colonization at 14 da and highlighted a reduction in *Escherichia coli* at different intestinal sites while *Clostridium* spp. showed a significant increase. Using LEfSe, we also noted that non-colonized TEG1 cecal microbiota show a greater abundance of *Enterobactericeae* compared to *C*. *jejuni*-colonized TEG2 at 2 dpi with relative increases in the abundance of *Clostridia* in the colonized birds (TEG2). The relative increase in the abundance of the *Enterobactericeae* was short lived with no significant differences between the age-matched samples from the non-infected group thereafter (Additional file 6).

In response to *Campylobacter* colonization, we observed variable shifts in the abundance of members of the *Clostridiales*, which are largely unclassified but feature members of the *Clostridiaceae*, *Lachnospiraceae*, and *Ruminococcaceae* families based on the consensus taxonomies. Increases in the abundance of clostridial species have been noted in association with experimental *C*. *jejuni* colonization previously [25, 54] and have been postulated to arise due to the *Campylobacter* acting as a hydrogen sink that would improve growth of clostridial organisms and their competitive standing through increased fermentation, leading to increased organic acid production that can be used by the campylobacters as an energy source [55]. However, several clostridial OTUs show greater abundance in the absence of *C*. *jejuni*, most notably *Clostridium* XIVa that feature in the analyses of the TEG and TLG experiments, and as major butyrate-producing bacteria play a key role in maintaining metabolic and immune functions in the gut [56].

**Fig. 8:**
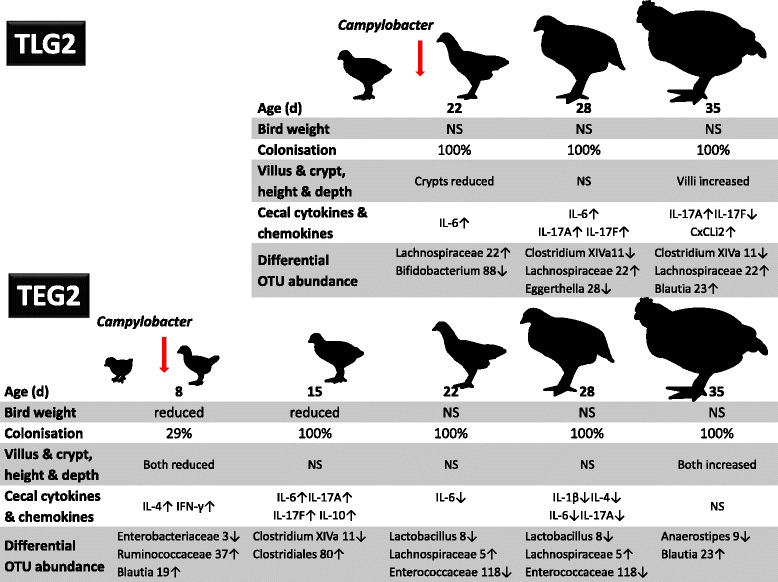
Summary of age-dependent differences between *C*. *jejuni*-colonized broiler chickens and non-infected controls. Time dependent reductions in the mean weights of *Campylobacter*-infected chickens are indicated. Relative increases in the cecal cytokine/chemokine expression of *C*. *jejuni*-colonized birds compared to age-matched non-colonized control birds are marked by up arrows (↑), and down arrows indicate decreases in cytokine/chemokine expression (↓). Representative members of the cecal microbiota showing greater differential abundances between age-matched *C*. *jejuni*-colonized birds (↑) and non-colonized controls (↓) are indicated by their consensus genera and corresponding abundance rank identifying OTU numbers. NS indicates no significant differences between *Campylobacter*-colonized birds and non-infected controls

It may be argued that variations in the abundance of the *Clostridiales* are a consequence of whether they benefit to the same degree from the bourgeoning *C*. *jejuni* population or show a relative reduction in abundance due to competition for alternative resources. These differential responses may also be driven by the prevailing chicken immune responses provoked by the *C*. *jejuni* colonization. For example, the late group will have to contend with pro-inflammatory cytokine and chemokine production in the ceca while the early group will have returned to levels similar to the non-infected group. *Ruminococcus* spp. OTUs identified from mature chicken cecal contents have been correlated with increases in IL-1β and IL-6 independent of any external microbial treatment [57], and therefore, any observed difference in abundance could represent a response to changing the immune status of the bird rather than a result of any direct interaction with a new member of the microbiota. Reductions in the abundance of *Clostridium *XIVa OTU0011 in the *C*. *jejuni*-colonized birds notably coincide with the peak Th17 pro-inflammatory responses that relate to the time of exposure in the TEG and TLG experiments (Fig. 8).

Microbial communities from fecal samples of *C*. *jejuni*-colonized commercial chickens at slaughter are reported to show increases in the abundance of *Streptococcus* and *Ruminococcaceae* and decreases in the abundance of *Lactobacillus* and *Corynebacterium* [55]. Notwithstanding that *Lactobacillus* are reported to be significantly over-represented in fecal samples compared with cecal content [57], we also found a relative decrease in the abundance of *Lactobacillus* spp. OTU0008 in the ceca of TEG2 *C*. *jejuni*-colonized birds. *Lactobacillus* spp. OTU0008 becomes significantly reduced at 16 dpi in the early colonized birds. This specific shift in the microbiota occurs after Th17 pro-inflammatory response in TEG2 when relative IL-6 expression is reduced and appears to represent a change in the microbiota driven by *C*. *jejuni* populations becoming established and tolerated (summarized in Fig. 8). In the late challenge where the Th17 response persists until the end of the study, the abundance of *Lactobacillus* spp. OTU0008 is not significantly changed. *Lactobacillus* spp. are established probiotics and have been proposed as feed additives to reduce the *C*. *jejuni* colonization of chickens [58–61]. If *C*. *jejuni* and *Lactobacillus* spp. OTU0008 compete for a similar niche and/or resource, then our observations could provide a basis for the inclusion of similar or better competing *Lactobacillus* ssp. in feed post programmed pro-inflammatory challenges such as those posed by vaccination. Inclusion would also have to minimize any potential negative impact on performance observed previously [2, 3], although it should be noted that at least one species of *Lactobacillus* spp. has been proposed to enhance the performance of broiler chickens [62].

## Conclusions

We have demonstrated specific increases in cytokine/chemokine expression that are consistent with a Th17 response to *C. jejuni* colonization for early and late infection experiments. However, the outcomes for the cytokine/chemokine responses differ with respect to the age of infection in that the early colonized birds return to levels not distinguishable from age-matched non-infected birds, whereas the later infection continues the show elevated IL-17A responses until the end of the study (summarized in Fig. 8). These differences do not result in lower *Campylobacter* colonization levels at the end of the study. It is evident that a sudden shift in microbiota, caused by the introduction and colonization of a highly successful enteric bacteria, would elicit an immune response but the response in itself is not necessarily an indication of pathogenic behavior. It has been suggested that the complex relationship that permits persistent, high-level cecal colonization of *C*. *jejuni* in its avian host without obvious pathology is a result of inefficiency within the chicken immune system combined with mechanisms that redirect the response toward tolerance [16]. Our data would suggest there are a range of age-dependent chemokine/cytokine responses that are targeted to the levels of colonization, which collectively drive shifts in the resident microbial communities.

## Additional files

Additional file 1: Mean weights of the broiler chickens from each experimental group. The mean live weights (SEM) of the chickens are plotted against the days of age for all experimental groups with the performance target weights for Ross 308 broiler chickens. TLG1—non-colonized control group for the late colonization experiment; TLG2—birds colonized with *C*. *jejuni* at day 20 for the late colonization experiment; TEG1—non-colonized control group for the early colonization experiment; TEG2—birds colonized with *C*. *jejuni* at day 6 for the early colonization experiment. (PDF 316 kb)

Additional file 2: Images of ileum H and E stained sections. Sections from non-infected control birds at 8 da (A), 22 da (B) and 35 da (C). Sections from *Campylobacter* infected birds in TEG2 at 2 dpi (D), 8 dpi (E) 15 dpi da (F) 28 dpi (G). The bars represent 200 μm. (TIF 8153 kb)

Additional file 3: The relative abundances 16S rRNA gene sequences of the most abundant phyla from the chicken ceca. The total read counts and the relative abundances are expressed as a percentage of the total reads for the most abundant taxonomic phyla discriminated at each sampling point over the rearing period of 35 days. (PDF 139 kb)

Additional file 4: PCoA plot of Bray-Curtis indices for the cecal microbiota of TEG. Bray-Curtis indices indicate the microbiota of birds exposed to *Campylobacter* at 6 da was different from uninfected birds at 2, 16 and 22 days post-infection by AMOVA (2 dpi; *p* = 0.026, 16 dpi; *p* = 0.039, 22 dpi; *p* = 0.003). R^2^ = 0.7; subsample = 16,319. (PDF 94 kb)

Additional file 5: Coliform and Lactic acid bacterial counts from cecal contents. Bar charts show log_10_ CFU/g intestinal content for coliform and lactic acid bacteria counts in: A, TLG1 and TLG2 birds and B, TEG1 and TEG2 birds. (PDF 229 kb)

Additional file 6: Differential abundance of members of the cecal microbial communities in the development of TEG *C. jejuni* colonized and non-colonized broiler chickens. Histogram of the LDA scores computed for features differentially abundant between *C. jejuni* colonized broiler chickens (denoted as “inf” by red bars) and non-colonized birds (denoted as “un” by green bars) over a 35 day rearing period. LEfSe identifies which clades amongst those detected as statistically differential will explain the greatest differences between the communities. OTUs represent individual sequences identified using BLASTn searches of type cultures with a BLAST identity ≥99%, and higher consensus taxanomic levels are indicated as _f family, _o order and _c class. Non-colonized birds were administered with 0.1 ml of carrier (MRD) by oral gavage at 6 da and colonized birds were with administered 10^7^ CFU *C. jejuni* strain HPC5 in 0.1 ml MRD at 6 da. Seven birds were sacrificed from each group at days 8, 15, 22, 28 and 35 from which cecal digesta were collected and total DNAs extracted in preparation for bacterial 16S rRNA gene analysis of the bacterial communities. (PDF 1539 kb)

## Abbreviations

AMOVA: Analysis of molecular variance
da: Days of age
dpi: Days post infection
FCR: Feed conversion ratio
OTU: Operational taxonomic unit
PCoA: Principal coordinate analysis
TEG: Trial early group
TLG: Trial late group
