# Supplementary material for: The effect of the timing of exposure to Campylobacter jejuni on the gut microbiome and inflammatory responses of broiler chickens
Source: Microbiome. 2018 May 12;6:88. doi: 10.1186/s40168-018-0477-5 (PMC5948730; doi:10.1186/s40168-018-0477-5)
Supplement: Supplementary file 1 — Mean weights of the broiler chickens from each experimental group. The mean live weights (SEM) of the chickens are plotted against the days of age for all experimental groups with the performance target weights for Ross 308 broiler chickens. TLG1—non-colonized control group for the late colonization experiment; TLG2—birds colonized with C. jejuni at day 20 for the late colonization experiment; TEG1—non-colonized control group for the early colonization experiment; TEG2—birds colonized with C. jejuni at day 6 for the early colonization experiment. (PDF 316 kb) [file 40168_2018_477_MOESM1_ESM.pdf]

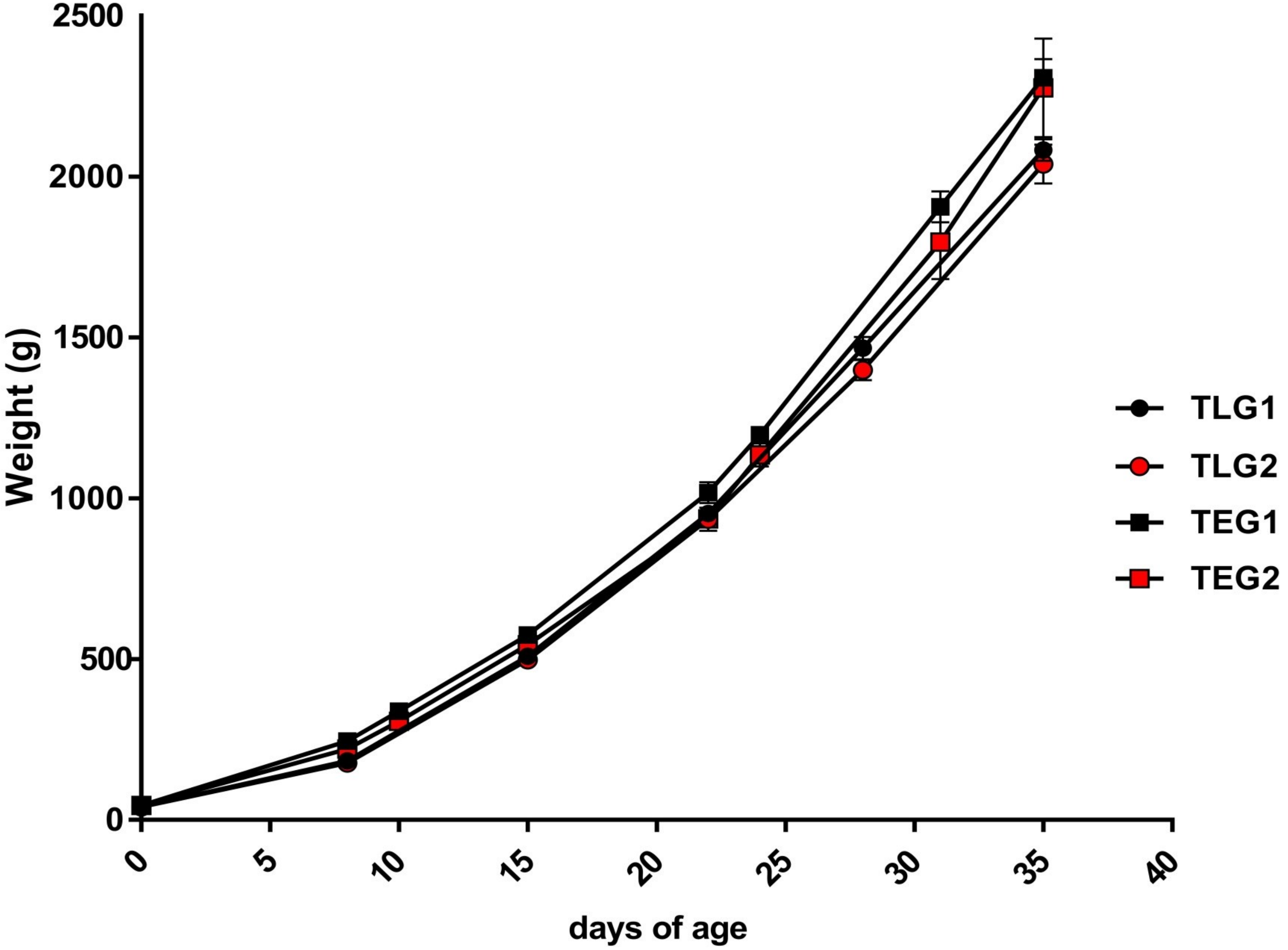

Additional File 1. Mean weights of the broiler chickens from each experimental group.
The mean live weights and SEM of the chickens are plotted against the days of age for all experimental groups with the performance target weights for Ross 308 broiler chickens. TLG1 – non-colonized control group for the late colonization experiment; TLG2 – birds colonized with C. jejuni at day 20 for the late colonization experiment; TEG1- non-colonized control group for the early colonization experiment; TEG2 – birds colonized with C. jejuni at day 6 for the early colonization experiment.
