# Supplementary figures and images for: The effect of the timing of exposure to Campylobacter jejuni on the gut microbiome and inflammatory responses of broiler chickens
Source: Microbiome. 2018 May 12;6:88. doi: 10.1186/s40168-018-0477-5 (PMC5948730; doi:10.1186/s40168-018-0477-5)

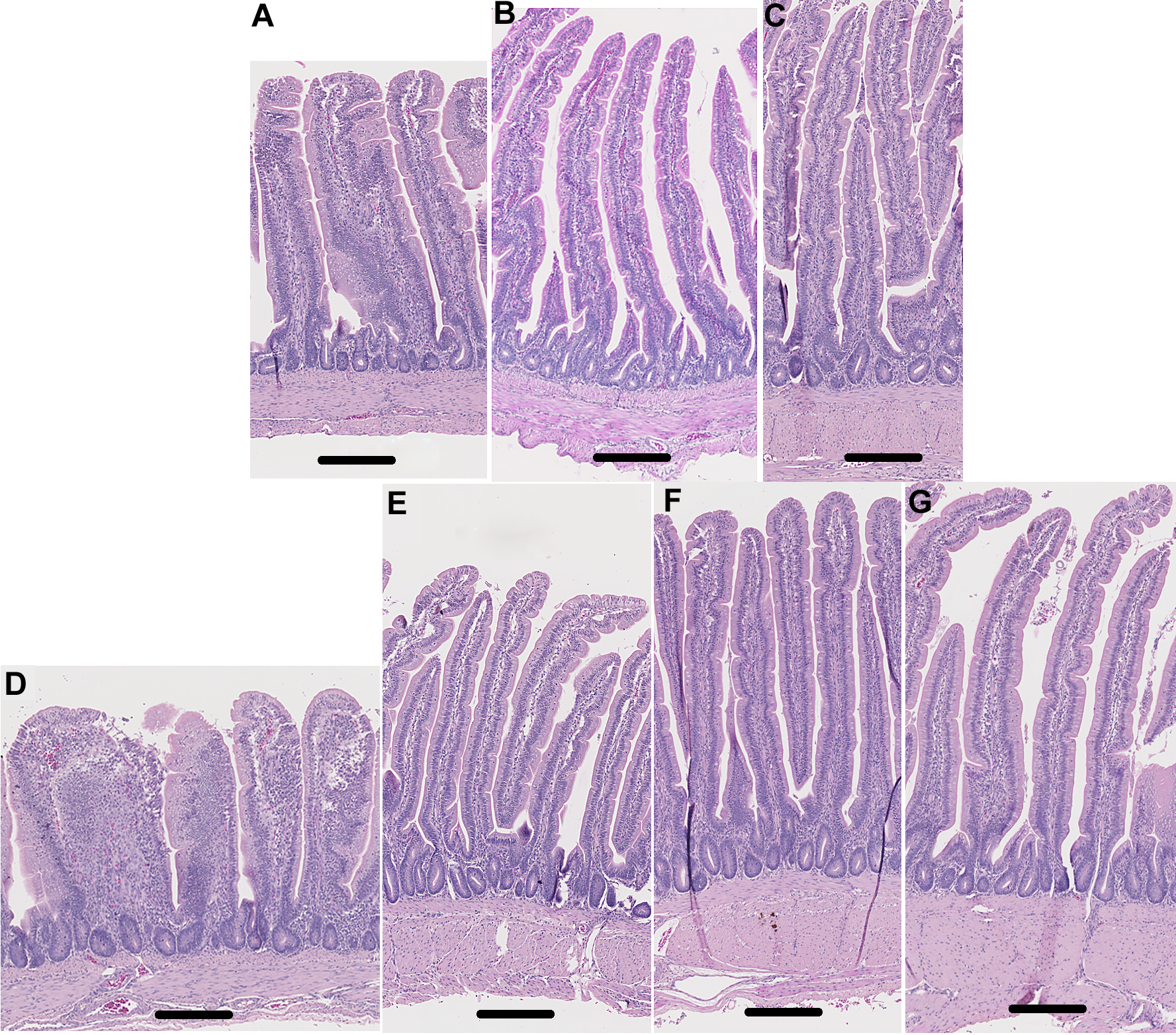

Supplement: Supplementary file 2 — Images of ileum H and E stained sections. Sections from non-infected control birds at 8 da (A), 22 da (B) and 35 da (C). Sections from Campylobacter infected birds in TEG2 at 2 dpi (D), 8 dpi (E) 15 dpi da (F) 28 dpi (G). The bars represent 200 μm. (TIF 8153 kb) [file 40168_2018_477_MOESM2_ESM.tif]

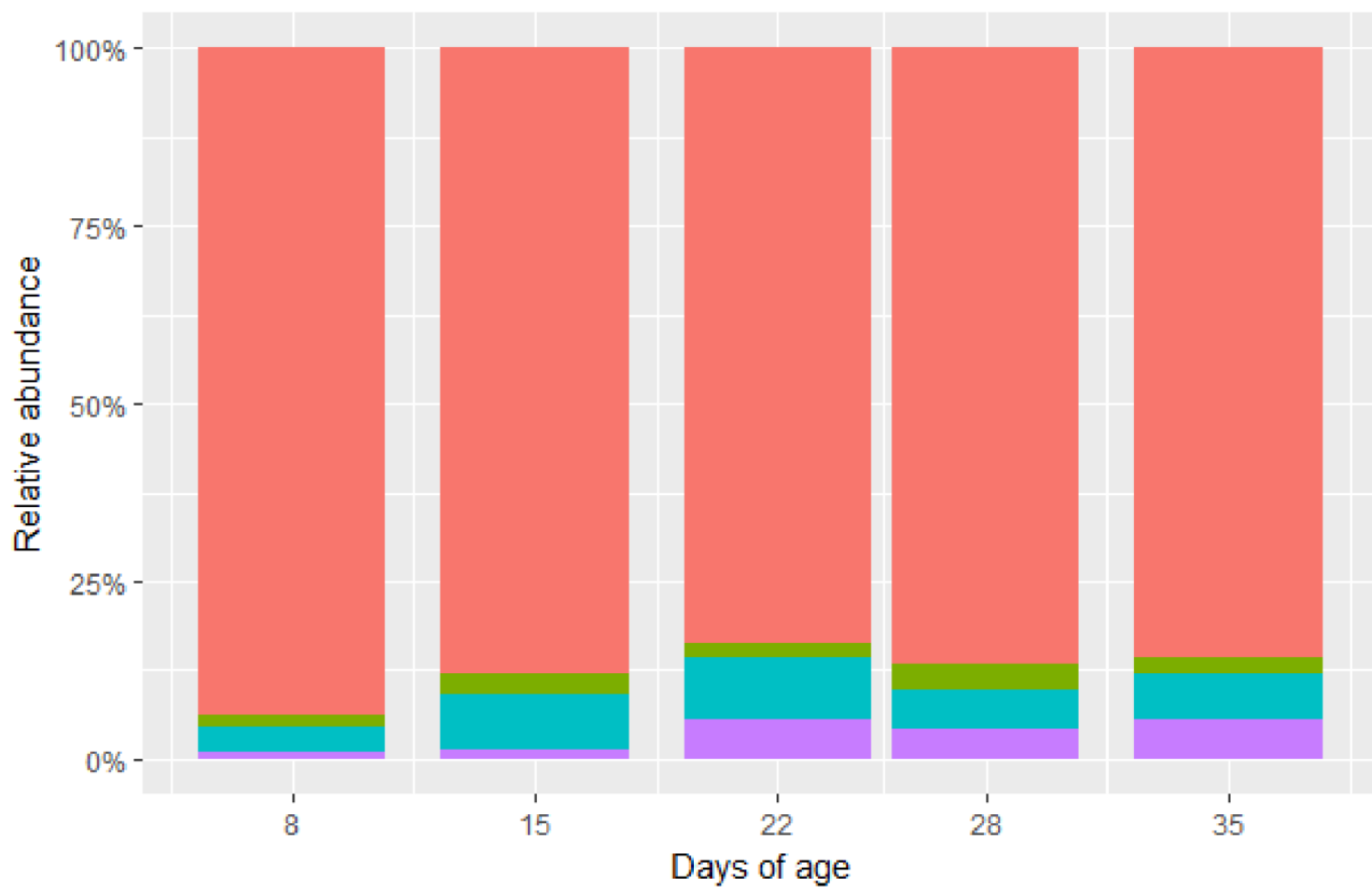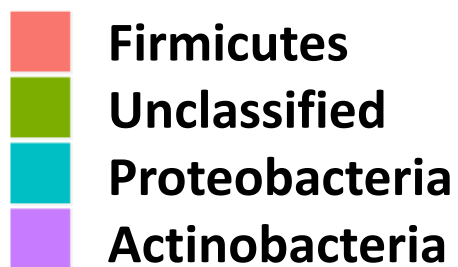

Supplement: Supplementary file 3 — The relative abundances 16S rRNA gene sequences of the most abundant phyla from the chicken ceca. The total read counts and the relative abundances are expressed as a percentage of the total reads for the most abundant taxonomic phyla discriminated at each sampling point over the rearing period of 35 days. (PDF 139 kb) [file 40168_2018_477_MOESM3_ESM.pdf]

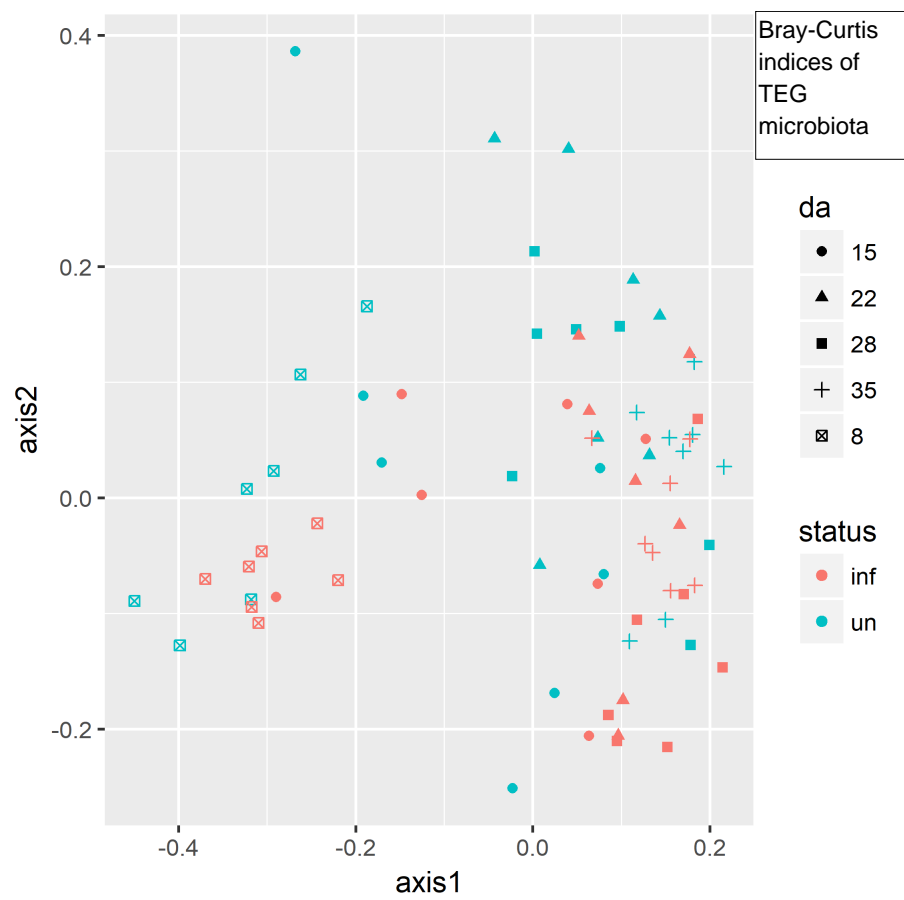

Supplement: Supplementary file 4 — PCoA plot of Bray-Curtis indices for the cecal microbiota of TEG. Bray-Curtis indices indicate the microbiota of birds exposed to Campylobacter at 6 da was different from uninfected birds at 2, 16 and 22 days post-infection by AMOVA (2 dpi; p = 0.026, 16 dpi; p = 0.039, 22 dpi; p = 0.003). R2 = 0.7; subsample = 16,319. (PDF 94 kb) [file 40168_2018_477_MOESM4_ESM.pdf]

inf

un

TEG day 8

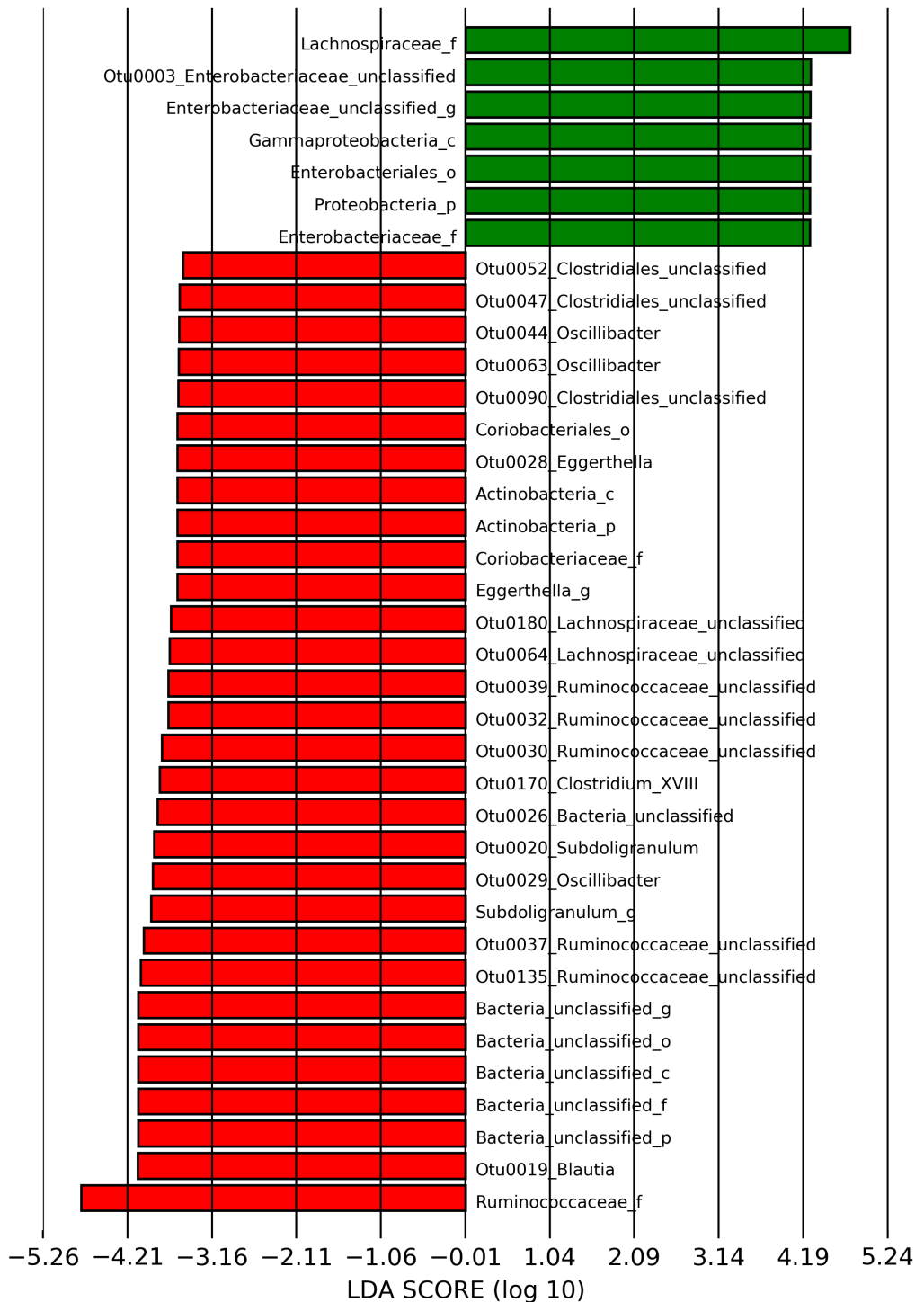

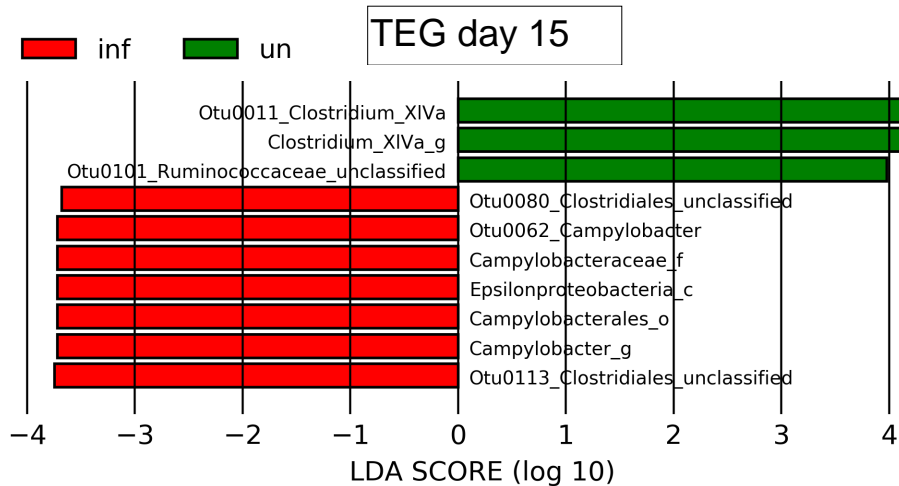

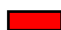

inf

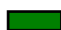

un

TEG day 22

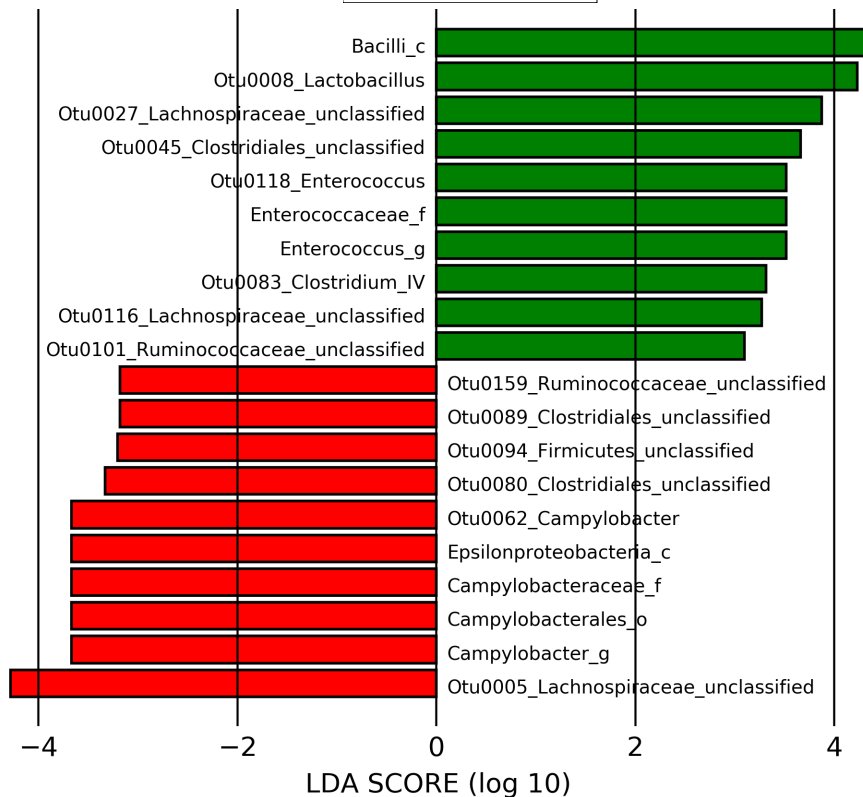

inf

un

TEG day 28

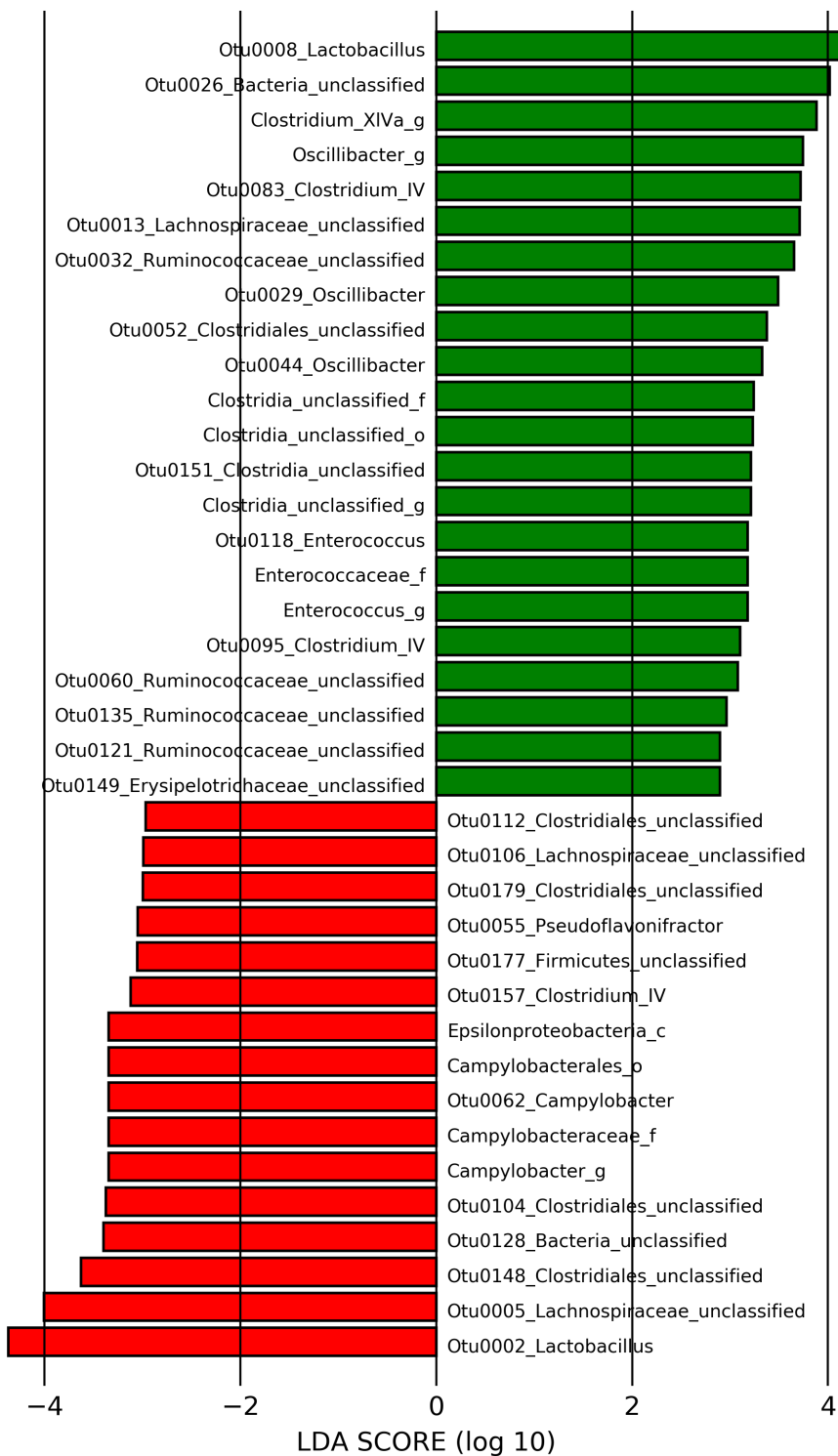

TEG day 35

inf un

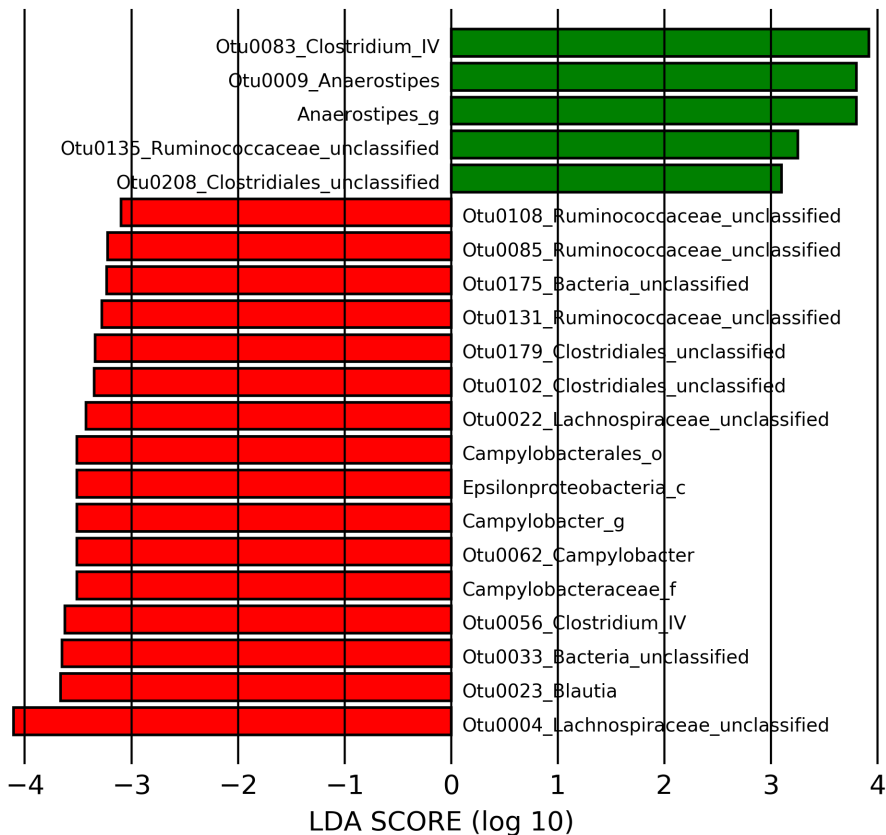

Supplement: Supplementary file 6 — Differential abundance of members of the cecal microbial communities in the development of TEG C. jejuni colonized and non-colonized broiler chickens. Histogram of the LDA scores computed for features differentially abundant between C. jejuni colonized broiler chickens (denoted as “inf” by red bars) and non-colonized birds (denoted as “un” by green bars) over a 35 day rearing period. LEfSe identifies which clades amongst those detected as statistically differential will explain the greatest differences between the communities. OTUs represent individual sequences identified using BLASTn searches of type cultures with a BLAST identity ≥99%, and higher consensus taxanomic levels are indicated as _f family, _o order and _c class. Non-colonized birds were administered with 0.1 ml of carrier (MRD) by oral gavage at 6 da and colonized birds were with administered 107 CFU C. jejuni strain HPC5 in 0.1 ml MRD at 6 da. Seven birds were sacrificed from each group at days 8, 15, 22, 28 and 35 from which cecal digesta were collected and total DNAs extracted in preparation for bacterial 16S rRNA gene analysis of the bacterial communities. (PDF 1539 kb) [file 40168_2018_477_MOESM6_ESM.pdf]
