# Supplementary material for: The effect of the timing of exposure to Campylobacter jejuni on the gut microbiome and inflammatory responses of broiler chickens
Source: Microbiome. 2018 May 12;6:88. doi: 10.1186/s40168-018-0477-5 (PMC5948730; doi:10.1186/s40168-018-0477-5)
Supplement: Supplementary file 5 — Coliform and Lactic acid bacterial counts from cecal contents. Bar charts show log10 CFU/g intestinal content for coliform and lactic acid bacteria counts in: A, TLG1 and TLG2 birds and B, TEG1 and TEG2 birds. (PDF 229 kb) [file 40168_2018_477_MOESM5_ESM.pdf]

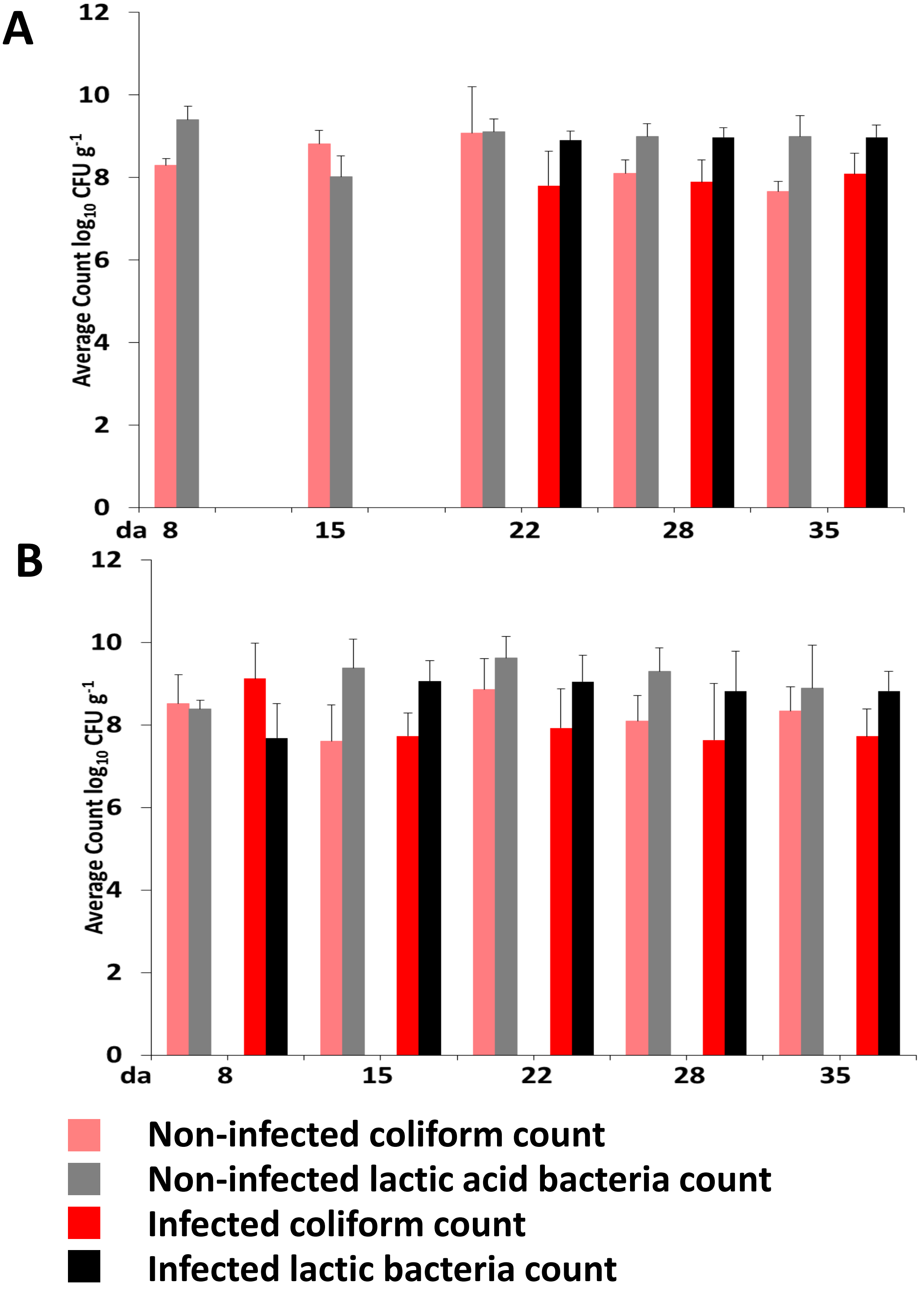

Additional File 4. Coliform and lactic acid bacteria counts in the cecal lumen. Error bars are standard deviations. A) Early cohort (TEG) infected at 6 da; B) Late cohort (TLG) infected at 20 da.
